# DefNEtTrp: An Iron Dual Chelator Approach for Anticancer Application

**DOI:** 10.1021/jacsau.4c00774

**Published:** 2024-12-04

**Authors:** Israel Rodríguez, Carmen Acosta, Christopher Nieves-Escobar, Estelle Strangmark, Oscar Claudio-Ares, Adriana I. Vargas Figueroa, Alexandra M. Soto-Millán, Aixa M. Orta-Rivera, Andrei V. Astashkin, Arthur D. Tinoco

**Affiliations:** † Department of Chemistry, University of Puerto Rico, Río Piedras Campus, Río Piedras, Puerto Rico 00931, United States; ‡ Department of Chemistry and Biochemistry, The University of Arizona, Tucson, Arizona 85721-0041, United States

**Keywords:** dual chelator, iron targeting, intracellular metal binding, metals in medicine, apoptosis and ferroptosis

## Abstract

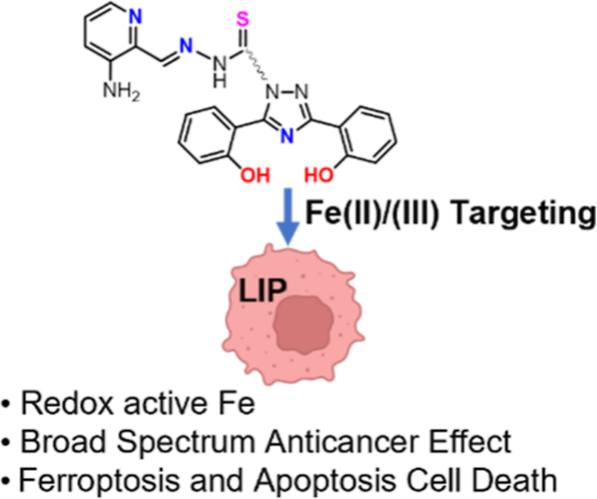

Targeting iron metabolism has emerged as a novel therapeutic strategy for the treatment of cancer. As such, iron chelator drugs are repurposed or specifically designed as anticancer agents. Two important chelators, deferasirox (Def) and triapine (Trp), attack the intracellular supply of iron (Fe) and inhibit Fe-dependent pathways responsible for cellular proliferation and metastasis. Trp, in particular, forms a redox active ferrous complex that inactivates the Fe-dependent ribonucleotide reductase (RNR), responsible for DNA replication. Building on recent efforts to employ intracellular Fe chelation for anticancer therapy, this work aimed to develop the Fe dual chelator ligand DefNEtTrp, consisting of the Def and Trp moieties, to exploit their high affinity Fe(II/III) binding and redox modulation. Using UV–vis spectroscopy, EPR spectroscopy, ESI and MALDI-TOF mass spectrometry, and cyclic voltammetry analyses, DefNEtTrp is shown to retain its Fe binding at both chelator moieties and generate a redox active Fe(III) complex Fe_3_(DefNEtTrp)_2_ featuring a reduction potential (*E*_1/2_ = +0.103 V vs normal hydrogen electrode) within the biological window. Screened against different cancer cell line types, DefNEtTrp exhibits potent and broad-spectrum antiproliferative and cell death behavior. Its cytotoxicity (IC_50_ 0.77 ± 0.06 μM) is superior to that of unconjugated Def and Trp ligands (IC_50_ 2.6 ± 0.15 μM and 1.1 ± 0.04 μM, respectively) in single-compound and combination treatments and is selective toward cancer cells. The cell death mechanism of the dual chelator is assessed in the context of intracellular labile Fe binding and was found to induce both apoptosis and ferroptosis.

## Introduction

During the COVID-19 pandemic, standard cancer diagnostic pathways were disrupted—with screenings for breast, colon, prostate, and lung cancers lowered by 85%, 75%, 74%, and 56%, respectively, in the US.^1^ Due to the underdiagnosis of cancers, the cases discovered after the height of the pandemic are likely to be more advanced and with worsened prognoses especially for communities of color.^2^ In response to this crisis, many research efforts are directed at targeting the hallmarks of cancer,^3^ focusing on pathways mediating cell growth, proliferation, and metastasis.

Essential metals such as iron play a key mechanistic role in these pathways. Cancer cells heavily rely on Fe for their characteristically rapid rate of cell division.^4^ They have far higher levels of serum transferrin (sTf) receptors than healthy cells do, indicating that they have a higher dependence on Fe, the metal that sTf transports from the blood into the cell.^5^ This results in elevated Fe influx and levels of the intracellular labile Fe pool (LIP), necessary to metalate the Fe-dependent enzymes/proteins for rapid cell division.^4,6,7^ Human ribonucleotide reductase (RNR), the rate-limiting enzyme of DNA synthesis, requires Fe in order to catalyze the formation of deoxyribonucleotides from ribonucleotides^8^ and it is overexpressed in cancer cells. An important mode of attacking the viability of cancer cells is targeting Fe bioavailability via the use of Fe-chelating ligands.^9,10^ For this reason, chelator drugs designed to treat iron overload diseases like the FDA-approved deferasirox (brand name Exjade) are being repurposed to explore their anticancer potential.^11^ Other chelator drugs, for instance, triapine,^12^ are designed with the specific intent to be used as anticancer agents.^13−15^ While some chelators can operate extracellularly by blocking cellular Fe uptake, others work intracellularly by scavenging cytosolic Fe from the LIP and disrupting the activation of Fe-dependent enzymes and also triggering Fe-centric toxicity such as ferroptosis.^16−18^

Our laboratory introduced a transmetalation approach to target intracellular Fe.^19−21^ Titanium(IV) complexes with high affinity Fe(III) chelators as ligands were developed to synergize intracellular Fe chelation with the cytotoxic potential of Ti(IV). These complexes undergo induced dissociation by transmetalating Ti(IV) for Fe(III) and are expected to operate only in the intracellular environment by reacting with the LIP.^19−21^ The Ti(deferasirox)_2_ complex exhibits RNR inhibitory capability contributed by both Ti(IV) and the chelator.^21^ Ti(IV) could decrease the nucleotide substrate pool by coordinating to them and even cleaving them by phosphate hydrolysis.^21^ In binding Fe(III), deferasirox (Def also known as DFX) could inhibit the activation of the RNR enzyme at its R2 subunit. Labile Fe(II) binds to a di-iron cluster site in the R2 subunit via oxidative addition, and this process generates an important tyrosyl radical located nearby. This tyrosyl radical activates the RNR by traveling to the R1 catalytic cysteine group, priming the initiation of ribonucleotide reduction. Def could also form a possible redox active Fe monoDef species that may lead to the reduction of the RNR R2 tyrosyl radical and inactivate the enzyme.^21^ In Jurkat cells, Ti(Def)_2_ arrests the cell cycle at the S phase, indicative of suppressed DNA replication likely from RNR inhibition.^21^

**Scheme 1:**
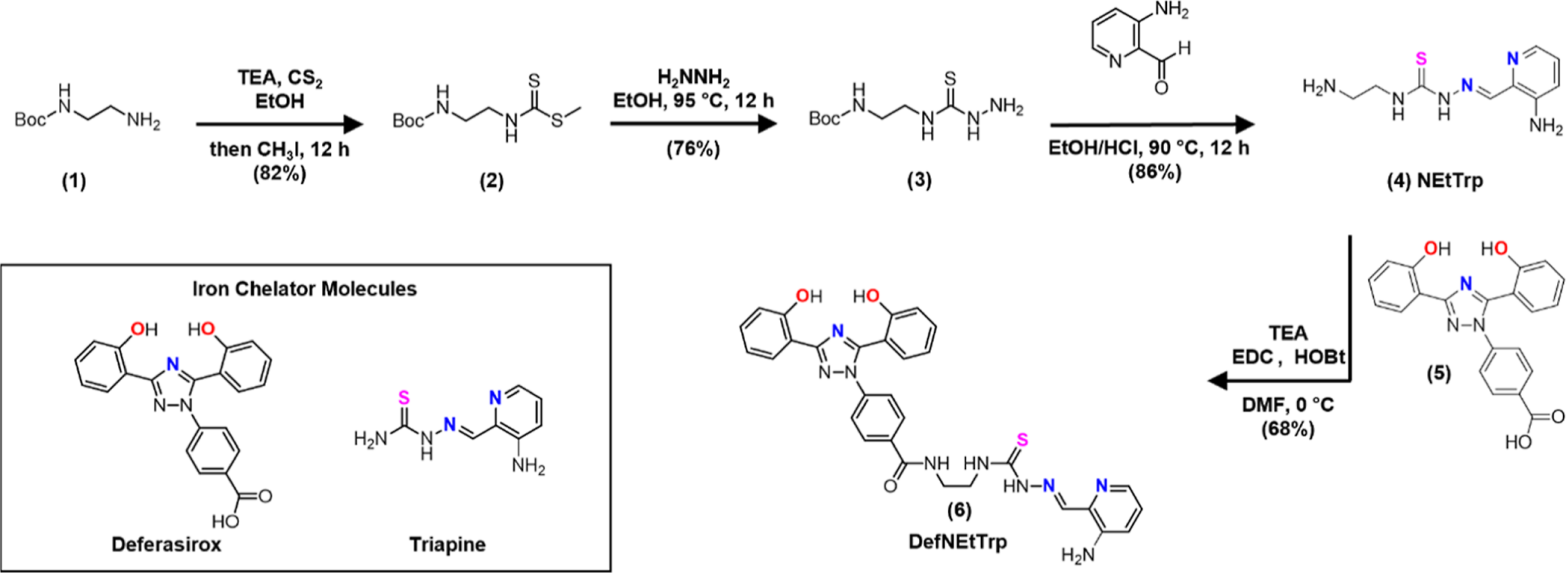
Synthetic Route of DefNEtTrp (**6**) The Fe coordinating atoms are highlighted in color.

This current study seeks to expand the tools available for anticancer drug design centered on targeting intracellular labile Fe by enhancing the chelation template to create a dual chelator ligand. This ligand was designed to feature hard and soft Lewis base chelators to facilitate effective Fe(II)/Fe(III) binding. To this end, a dual chelator consisting of a covalent conjugation of the hard Lewis base ONO chelator Def and of the soft/intermediate Lewis base NNS triapine (Trp also known as 3-AP) moieties was synthesized (Scheme 1). Triapine was judiciously selected to expand on the already established utility of the Def moiety^20,21^ in this design for two important reasons. The intracellular LIP consists of an equilibrium between Fe(II) and Fe(III) but Fe(II) species predominate because of the cellular reducing environment.^22−24^ Unlike Def, Trp features both high Fe(II) and Fe(III) affinity at pH 7.4 (cytosolic pH is 7.1–7.2)^25^ and thus would enable the dual chelator conjugate to more broadly interact with the LIP. Trp can form the redox active Fe(II)(Trp)_2_ species within cells, which is an effective reductant of the RNR R2 tyrosyl radical.^26^ Molecular docking has shown that Trp can bind near the RNR R2 di-iron cluster site^27^ revealing a route by which the Fe(II)(Trp)_2_ species may come into close contact with the tyrosyl radical. Trp has also been observed to induce the iron-dependent cell death pathway ferroptosis. In this pathway, cell death occurs because of the formation of excessive lipid peroxidation due to an increase in intracellular highly redox active Fe species and corresponding significant increase in reactive oxygen species (ROS).^28^ In this work, we synthesize the dual chelator deferasirox *N*-ethyleneamine triapine (DefNEtTrp) and explore its Fe(III) complexation. The Fe-binding capability of the dual chelator and its associated redox activity are assessed in the context of its broad-spectrum and selective anticancer activity and highly potent cytotoxicity, which is superior to the behavior of the unconjugated chelators in individual and combined treatments.

## Results and Discussion

### Synthesis and Characterization of the Dual Chelator Ligand DefNEtTrp

A facile synthetic route was used to conjugate the two Fe chelators Def (**5**) and Trp with an ethylenediamine linker, resulting in a high-yield product. The synthesis of DefNEtTrp (**6**) was accomplished through a four-step synthetic route (see Scheme 1) adapting a literature protocol.^29^ First, nucleophilic addition of *N*-Boc-ethylenediamine to carbon disulfide followed by methylation with iodomethane was performed to yield methyl-*N*-(2-*tert*-butoxycarbonylaminoethyl)-dithiocarbonate (**2**). Second, the nucleophilic addition–elimination reaction with hydrazine monohydrate yielded thiosemicarbazide (**3**). *N*-Ethyleneamine triapine dihydrochloride (NEtTrp·2HCl) (**4**) was then obtained through a condensation reaction between (**3**) and 3-amino-picolinaldehyde in ethanolic HCl. These conditions generated quality X-ray diffracting single crystals of (**4**) (Figure S1 and Table S1; CCDC deposition number 2216216). The DefNEtTrp (**6**) conjugate compound was obtained via carbodiimide-mediated amide coupling of (**4**) and Def (**5**). Each of the intermediates was confirmed by ^1^H nuclear magnetic resonance (NMR) spectroscopy (peaks are detailed in the experimental section and spectra are included in Supporting Information Figures S2–S4).

DefNEtTrp was characterized by electrospray ionization-mass spectrometry (ESI-MS), ^1^H and ^13^C NMR, and C,H,N elemental analysis. The measured mass distribution of the compound matches the theoretical distribution of {(H^+^)[C_30_H_27_N_9_O_3_S]}^+^, (*m*/*z* = 594.23) (Figure S5). Additionally, ^1^H and ^13^C NMR data (Figures S6–S7) and C,H,N elemental analysis confirm the successful synthesis of the compound in high purity.

The lipophilicity of DefNEtTrp was characterized by measuring the partition coefficient (log *D*_pH7.4_) of the compound between 1-octanol and 1× PBS (pH 7.4) using the shake-flask method^30^ and compared with the value for Def and Trp also measured here experimentally. Due to the general low solubility of the compounds in either 1-octanol and water, stock solutions had to first be prepared in dimethyl sulfoxide (DMSO) and then diluted into the two cosolvents to a final 5% DMSO (v/v). The partition coefficients for Trp, Def, and DefNEtTrp were 0.255, 0.427, and 0.433, respectively. The lipophilic comparability of DefNEtTrp with Def suggests that it should be cell-permeable. It, however, is expected to exhibit poor oral druglikeness as it violates several of Lipinski’s rule of 5 including exceeding 500 g/mol in molecular weight, having more than 5 hydrogen bond donors and more than 10 hydrogen bond acceptors. Def and Trp,^31^ in contrast, are orally bioavailable. This assessment is supported by consulting the SwissADME Web site.^32^

### Characterization of the Fe(III) Complexation by DefNEtTrp (**6**)

**Figure 1:**
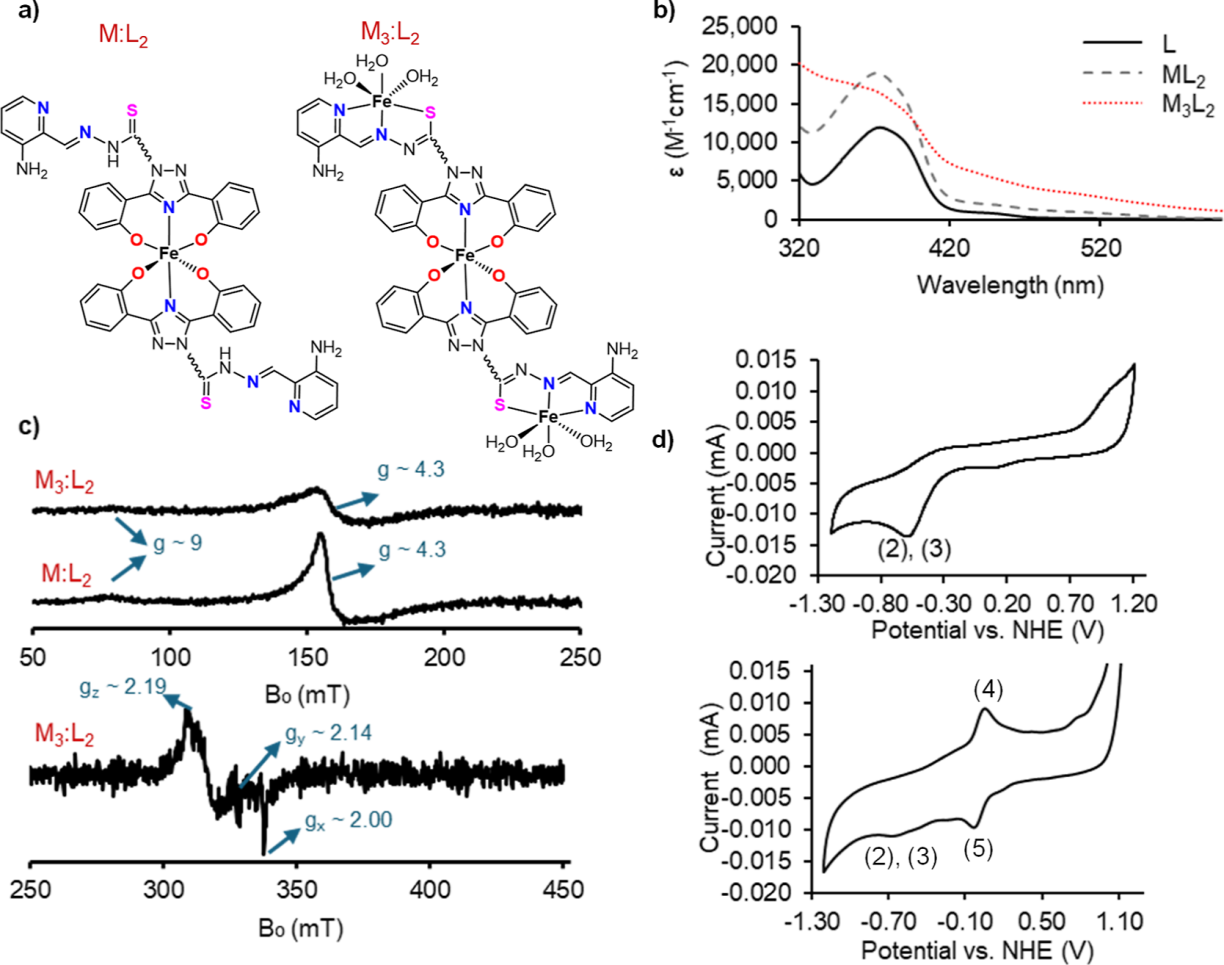
Characterization of the Fe(III) DefNEtTrp complexation. (a) Proposed structures for Fe(DefNetTrp)_2_ (**7**) and Fe_3_(DefNetTrp)_2_ (**8**). The Fe_3_(DefNetTrp)_2_ complex likely consists of a mixture of coordinated water and hydroxide molecules. (b) UV–vis spectra of DefNEtTrp (L; black), Fe(DefNEtTrp)_2_ (ML_2_; black dashes), and Fe_3_(DefNEtTrp)_2_ (M_3_L_2_; red dots). The extinction coefficient (ε) is normalized to DefNEtTrp concentration. (c) EPR spectrum of Fe(DefNetTrp)_2_ and Fe_3_(DefNEtTrp)_2_. Experimental conditions: microwave frequency, 9.442 GHz; magnetic field modulation amplitude, 0.5 mT; microwave power, 2 mW for high-spin Fe(III) (*S* = 5/2) detection, microwave power; 200 μW for low-spin Fe(III) (*S* = 1/2) detection; temperature: 77. (d) Cyclic voltammogram of 6 mM Fe(DefNetTrp)_2_ and Fe_3_(DefNetTrp)_2_.

Fe(III) complexation by DefNEtTrp was initially studied by reacting the ligand with Fe(III) dicitrate (Fe(Citrate)_2_), which is a labile Fe(III) complex. This reaction yielded the sodium salt of Fe(DefNEtTrp)_2_ (**7**) even with an excess of Fe(III) (Figure 1A). The compound was determined to be Na[Fe(C_60_H_50_N_18_O_6_S_2_)]·2H_2_O·2 CH_3_OH by C,H,N elemental analysis. While it is not readily soluble in water, an aqueous solution of it can be obtained by first dissolving it in a cosolvent like dimethylformamide (DMF), DMSO, or primary alcohols. The ultraviolet–visible (UV–vis) spectrum of the compound at pH 7.4 (50:50 DMSO:H_2_O) shows a LMCT absorbance at 450 nm (ε = 3200 M^–1^ cm^–1^ based on compound concentration) (Figure 1B). The n → π* absorbance at 375 nm (ε = 11,900 M^–1^ cm^–1^) increases due to Fe(III) coordination (ε = 18,100 M^–1^ cm^–1^ based on ligand concentration). The electrospray ionization mass spectrum (ESI-MS) confirms a 1:2 metal:ligand species (Figure S8). An EPR spectrum was collected to determine the Fe(III) coordination modality (Figure 1C). The spectrum is for a high-spin Fe(III) (*S* = 5/2) complex comparable to the Fe(deferasirox)_2_ spectrum we previously collected,^21^ indicating Fe(III) coordination to the ONO atoms of the deferasirox moiety with meridional arrangement of the DNT ligands (Figure 1A). It consists of general rhombic symmetry (*E*/*D* ≈ 1/3) containing a major line at *g* ≈ 4.3 belonging to the middle Kramers doublet and the weak feature at *g* ≈ 9 from the top and bottom Kramers doublets. The UV–vis spectrum for Fe(deferasirox)_2_**(9)** was collected at pH 7.4 (50:50 DMSO:H_2_O) for additional comparison purposes (Figure S9). It demonstrates two LMCT absorbances at 420 nm (ε = 5500 M^–1^ cm^–1^ based on compound concentration) and 500 nm (ε = 3400 M^–1^ cm^–1^ based on compound concentration), which is on the order of the absorbance intensity reported above for Fe(DefNEtTrp)_2_.

At pH 7.4, Def and Trp display a high affinity for Fe(III) but the affinity of Def is far higher (log β for Fe(III)(Trp)_2_ = 26.3 vs log β for Fe(III)(Def)_2_ = 36.9).^25,33^ Thus, it is reasonable that in interaction with DefNEtTrp, Fe(III) would preferentially bind to the Def moiety.^33^ To support this hypothesis, a pH-dependent speciation model was produced (Figure S10) for 50 μM Fe(III) in interaction with Def and Trp (both at 100 μM). This model reveals that at pH 7.4, Fe(III) exclusively binds Def as the Fe(Def)_2_^2–^ species, which holds true for micromolar levels of Fe(III) in general in which the metal:Def:Trp ratio is maintained at 1:2:2.

To study higher order Fe(III) binding by DefNEtTrp, Fe_2_(SO_4_)_3_ was used, which behaves more like an Fe(III) salt in an aqueous solution. In this experiment, 20 μM Fe(DefNEtTrp)_2_ (solution made by dissolving the isolated solid) was reacted with varying concentrations of Fe_2_(SO_4_)_3_ in separate solutions, up to 200 μM Fe(III) at pH 7.4 (50:50 (v/v) DMSO:H_2_O). These solutions were left to equilibrate for 6 h and then the UV–vis spectra were collected over the wavelength range of 300 to 600 nm at 25 °C. The spectra were deconvoluted with the ReactLab Equilibria software and fit to a simple model for the formation of a Fe_3_(DefNEtTrp)_2_ (**8**) species (eq 1, Figure S11). The log β value was determined to be 11.1, and the pH 7.4 relative log β value was determined to be 25.9. The UV–vis spectrum for Fe_3_(DefNEtTrp)_2_ shows a LMCT absorbance at 448 nm (ε = 11,000 M^–1^ cm^–1^ based on compound concentration) and at 512 nm (ε = 6400 M^–1^ cm^–1^ based on compound concentration). The n → π* absorbance at 374 nm (ε = 16,200 M^–1^ cm^–1^ based on ligand concentration) decreases relative to the same absorbance in Fe(DefNEtTrp)_2_ (Figure 1B).
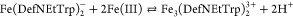
1

The synthesis of a Fe_3_(DefNEtTrp)_2_ species was performed by reacting DefNEtTrp with a stoichiometric excess of the Fe(III) salt FeCl_3_ in the pH range of 7 to 8. The product is a black-red solid that exhibits very low water solubility. Its proposed structure (Figure 1A) consists of Fe(III) bound meridionally by the Def groups and the other two Fe(III) ions bound at the Trp groups, with water molecules or hydroxides completing the remainder of the coordination sites. Scanning electron microscopy-energy-dispersive spectroscopy (SEM-EDS) data confirm the presence of Fe, C, N, O, and S atoms in the compound (Figure S12). The FTIR spectrum of the compound in the 1900 to 750 cm^–1^ region reveals vibration frequencies characteristic of DefNEtTrp but shifted due to metal coordination (Figure S13). An EPR spectrum was collected to determine the Fe(III) coordination (Figure 1C). The spectrum shows both high-spin (*S* = 5/2) and low-spin (*S* = 1/2) Fe(III). The high-spin signal is attributed to the Fe(III) complexation by the deferasirox moieties, with a general rhombic symmetry containing a major line at *g* ≈ 4.3 belonging to the middle Kramers doublet and the weak feature at *g* ≈ 9 from the top and bottom Kramers doublets. The low-spin signal is believed to be due to coordination by the triapine moiety, with a rhombic symmetry (*g*_*z*_ ≈ 2.19; *g*_*y*_ ≈ 2.14; *g*_*x*_ ≈ 2.00).

To further make sense of the Fe(III) coordination within the Fe_3_(DefNEtTrp)_2_ complex, 1:1 Fe:triapine (pH 2) and 1:2 Fe:triapine (pH 8) species were prepared in situ following well-characterized speciation studies, by reacting 1 mol equivalent of Fe(III) with 2.5 equiv of triapine and carefully equilibrating the solutions to their respective pH values.^25^ The UV–vis spectra for the two in situ prepared solutions collected at 50 μM Fe(III) exhibit two distinct species (Figure S14) and were corrected for excess ligand in the respective solutions. The UV–vis spectrum for the Fe(triapine) (**10**) (50:50 DMSO:H_2_O) shows a LMCT absorbance at 500 nm (ε = 3000 M^–1^ cm^–1^ based on compound concentration). The n → π* absorbance at 385 nm (ε = 16,500 M^–1^ cm^–1^ based on ligand concentration) increases due to Fe(III) coordination (ε = 21,600 M^–1^ cm^–1^ based on ligand concentration). This solution is light amber in color. The UV–vis spectrum for the Fe(triapine)_2_ species (**11**) shows two LMCT absorbances at 455 nm (ε = 12,800 M^–1^ cm^–1^ based on compound concentration) and 500 nm (ε = 3200 M^–1^ cm^–1^ based on compound concentration). The n → π* absorbance at 374 nm (ε = 16,200 M^–1^ cm^–1^ based on ligand concentration) decreases and is comparable to that of metal-free triapine. This solution is dark amber in color. The EPR spectra of the pH 2 and 8 species are very similar, revealing a low-spin (*S* = 1/2) Fe(III) species (Figure S15). The spectra reveal rhombic symmetry (*g*_*z*_ = 2.186; *g*_*y*_ = 2.137; *g*_*x*_ = 1.996) comparable to the low-spin Fe(III) signal in the EPR spectrum for Fe_3_(DefNEtTrp)_2_.

Positive ion MALDI-TOF MS of Fe_3_(DefNEtTrp)_2_ (the compound did not fly in the ESI-MS instrument) shows a peak with the highest *m*/*z* value at 1348.10 for the putative Fe_3_(DefNEtTrp)_2_ species (Figure S16). The Fe(DefNEtTrp)_2_ and Fe_2_(DefNEtTrp)_2_ species are also observed, likely from the ionization lability of the Fe ions at the Trp moieties during the data collection.

The Fe(III) redox behavior in Fe(DefNEtTrp)_2_ and Fe_3_(DefNEtTrp)_2_ provides coordination and activity insight. A cyclic voltammetric analysis of 6 mM concentration of both compounds (Figure 1D) was recorded in DMF solution with added hydroxide to approximate pH 7.4 (see experimental conditions) and compared with that of Fe(Def)_2_ (measured in this work) and Fe(Trp)_2_ collected under similar conditions.^27^ Fe(Def)_2_ undergoes an irreversible reduction to the Fe(II) form at *E*_pc_ = −0.60 V vs normal hydrogen electrode (NHE) (Figure S17). In the Fe(Def)_2_ complex, Fe(III) is bound with high affinity and is very solution-stable, particularly in an aqueous solution at pH 7.4. However, in its Fe(II) form, the Fe(deferasirox)_2_ complex appears to be unstable, resulting in the dissociation of Fe(II). Previous aqueous speciation studies have shown that Fe(II) partially dissociates from Def at pH 7.4 but is more stable at basic pH.^33^ Fe(Trp)_2_ has a reversible redox couple of *E*_1/2_ = +0.01 V vs NHE.^27^ As an intermediate/soft Lewis base, Trp has a high affinity for Fe(II) (log β for Fe(II)(Trp)_2_ = 22.55)^25^ at pH 7.4, whereas the hard Lewis base Def has a significantly lower affinity (estimated log β for Fe(II)(Def)_2_ = 14.0).^33^ Fe(DefNEtTrp)_2_ has an irreversible reduction at *E*_pc_ = −0.62 V vs NHE owed to the same solution behavior exhibited by Fe(Def)_2_ (eqs 2 and 3)
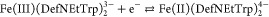
2
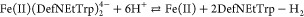
3

Fe_3_(DefNEtTrp)_2_ also exhibits this irreversible reduction at *E*_pc_ = −0.56 V versus NHE, characteristic of Fe(III) coordination to the Def moiety. In addition, it has a reversible redox couple of *E*_1/2_ = +0.103 V vs NHE with a peak potential separation of Δ*E*_p_ = 30 mV, indicative of a two-electron process (eqs 4 and 5).
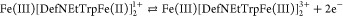
4
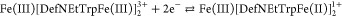
5

This redox couple is attributed to the two Fe(III) ions coordinated by the Trp moieties, which explains the two-electron process and suggests that Fe coordination in the +2 and +3 oxidation states by the Trp moiety remains intact under these solution conditions.

A recent study exploring Fe(II/III) complexation by Trp and Trp analogues revealed that complexes that exhibited a reversible redox couple less than *E*_1/2_ = 0.2 V vs NHE and not greater than *E*_1/2_ = 0.5 V vs NHE could effectively redox cycle in the cellular environment and reduce the tyrosyl radical of RNR R2.^34^ Thus, Fe complexation at the Trp moiety of DefNEtTrp can be expected to exhibit these properties. This finding is important in the context of the cytotoxic behavior exhibited by DefNEtTrp as later discussed.

The ability of DefNEtTrp to coordinate and remove Fe(III) bound to sTf, the main Fe(III) species in serum,^35^ was also explored. Fe(III)-bound sTf plays vital functions of regulating Fe homeostasis and bioavailability in blood and delivering the metal to all cells in the body. STf features two high affinity Fe(III) binding sites^36^ that undergo a conformational change after Fe(III) binding that limits solvent exposure^37,38^ and makes the metal extremely ligand-exchange-inert.^39^ The reaction of a near physiological concentration of Fe_2_-sTf (sTf is present in blood at 30 to 60 μM) with an equimolar amount of DefNEtTrp with respect to Fe(III) concentration was monitored over 3 days. The characteristic LMCT absorbance (λ_max_ 465 nm; ε = 5200 M^–1^ cm^–1^)^40^ was unchanged indicating no metal dissociation. As further confirmation, the reaction solutions were extensively dialyzed, and the protein concentration-normalized UV–vis spectra showed fully intact Fe_2_-sTf. This was also confirmed by Fe quantification via the ferrozine assay. As a potential anticancer therapeutic, this is a significant finding as the ligand would not be able to cause systemic toxicity by scavenging Fe from sTf and disrupting its normal biodistribution.

Collectively, the experimental results point to Fe(III) being able to bind dominantly at the Def group of DefNEtTrp in aqueous solution under physiologically relevant conditions when present as an Fe(III) complex. This provides insight into Fe(III) complexation by DefNEtTrp in reaction with the intracellular LIP, which consists of small molecular Fe(III) species.^41^

### Correlating Fe Binding to the Cytotoxicity of DefNEtTrp

DefNEtTrp was submitted to the National Cancer Institute (NCI) 60 human tumor cell line anticancer drug screen, of which 57 were actually tested (Table S2). The cell lines represent nine human cancers: leukemia, melanoma, lung, colon, central nervous system, ovarian, renal, prostate, and breast cancers. Due to its very promising broad-spectrum antiproliferative activity exhibited against the cell lines in a one-dose test of 10 μM administered for 48 h, DefNEtTrp was advanced to a five-dose assay (0.01, 0.1, 1, 10, and 100 μM) for 48 h and to a final repeated five-dose assay to verify reproducibility. Three-dose–response parameters were measured: 50% growth inhibition (GI_50_), total growth inhibition (TGI), and 50% cellular death (LC_50_). GI_50_ measures the concentration of a compound that induces 50% inhibition of the cells to reach maximal cell count relative to the initial cell total under no compound treatment conditions. TGI measures the compound concentration that retains the cells at the initial cell count used in the screen. LC_50_ measures the concentration that reduces (kills) 50% of the initial cell total. Table 1 tabulates the calculated averages of GI_50_ and TGI (the Supporting Information includes the full testing results including the LC_50_ values in Table S2 and the dose–response curves of the nine different panels of cell lines in Figure S18). DefNEtTrp was able to inhibit 50% cell growth (proliferation) of all cell lines at an average GI_50_ concentration of 1.2 μM. It showed the highest antiproliferative sensitivity to leukemia with a GI_50_ of 0.29 μM and the least sensitivity to renal cancer with a GI_50_ of 4.97 μM. Interestingly, the broad-spectrum antiproliferative behavior exhibited by DefNEtTrp is superior to the activity of triapine according to the information available in the NCI-60 database (Table S3; compound S759096). Trp had a higher average GI_50_ concentration of 3.5 μM relative to that of DefNEtTrp but did exhibit a better cytotoxic profile against renal cells. No NCI-60 database information was available for Def for the two day assay likely because this compound was not originally developed for anticancer purposes. DefNEtTrp induced total cell growth inhibition against 77% of the cell lines tested at an average TGI concentration of 8.5 μM. Of these cells, the lowest average TGI (1.78 μM) was observed for leukemia. DefNEtTrp also exhibits cell death capability. It induced 50% cell death against 14% of the tested cell lines at an average LC_50_ of 38.9 μM.

**Table 1:** Calculated Average 50% Growth Inhibition (GI_50_) and Tumor Growth Inhibition (TGI) of DefNEtTrp in μM Drug Concentration

cancer cell line type	GI_50_ (μM)	TGI (μM)^a^
leukemia	0.29	1.78
nonsmall cell lung	0.73	6.33
colon	0.87	12.22
CNS	0.50	12.43
melanoma	0.61	4.73
ovarian	1.12	9.82
renal	4.97	13.71
prostate	1.14	18.2
breast	0.45	4.84

a Average calculated for cell lines in which TGI was observed.

Given the virtually 100% antiproliferative behavior exhibited by DefNEtTrp against all leukemia cell lines in the NCI screen, a more in-depth analysis of its activity was performed against the leukemia Jurkat cell line as a case study. The viability of Jurkat cells was examined in a 72 h treatment with DefNEtTrp, Def, NEtTrp, and Trp, a 1:1 combination of Def and NEtTrp, and a 1:1 combination of Def and Trp. All compounds were examined in the concentration range of 100 nM to 20 μM. Table 2 provides the inhibitory concentration of the compounds that results in 50% of the maximal cell count under no treatment conditions (IC_50_ values; a number not relative to the initial cell count). Figure S19 shows the dose–response curves for the tested compounds and combinations. All conditions resulted in near 0% cell viability suggestive of antiproliferative and cell death capabilities in the concentration range examined. DefNEtTrp demonstrates a superior cytotoxic effect compared to Def, NEtTrp, and Trp in single-compound and combination treatments.

**Table 2:** IC_50_ Values of the Compounds Trp, NEtTrp, Def, and DefNEtTrp, 1:1 Combination of Def and NEtTrp, and 1:1 Combination of Def and Trp Treated against the Jurkat Cell Line for 72 h

compounds	IC_50_ (μM)
Trp	1.1 ± 0.04
NEtTrp	5.9 ± 0.29
Def	2.6 ± 0.15
DefNEtTrp	0.77 ± 0.06
1:1 combination of Def and NEtTrp	4.9 ± 0.18
1:1 combination of Def and Trp	3.1 ± 0.17

**Figure 2:**
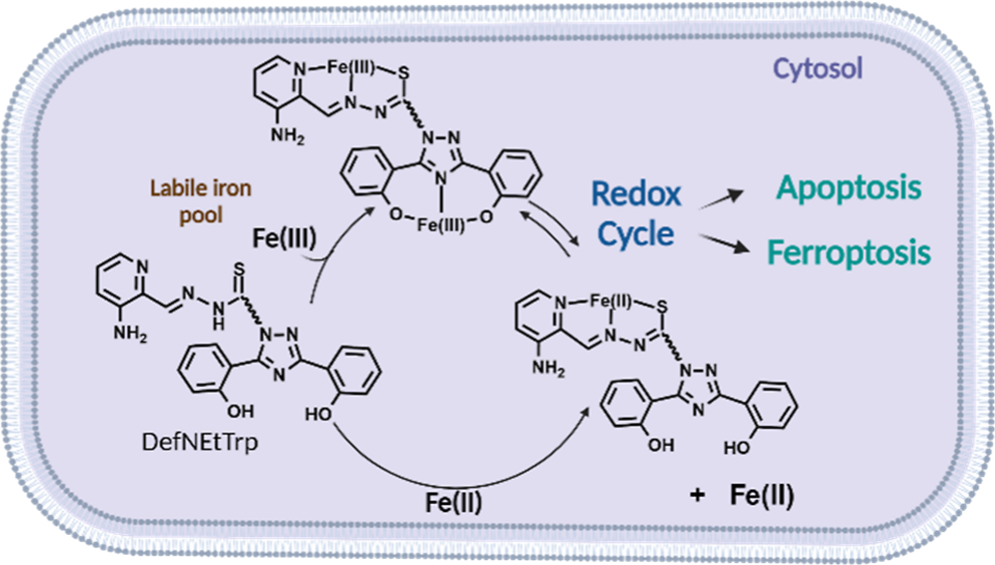
Proposed Fe-dependent cytotoxic mechanism of DefNetTrp. This image was created using BioRender.com.

The heightened potency of DefNEtTrp is believed to be due to the conjugation of the two chelators facilitating a synergistic redox activity. As already established, the Fe(III) coordination to the Trp moiety would be able to redox cycle and reduce the RNR R2 tyrosyl radical and inactivate the enzyme. In our previous study, we reported that redox active Fe(III) monoDef species could potentially form intracellularly.^21^ These species may be expected to readily reduce to Fe(II), release the Fe(II), and recycle by binding and reducing other Fe(III) ions. Thus, the dual chelator DefNEtTrp could result in simultaneous formation of Fe(monoDef) and Fe(monoTrp) species, producing a powerful reductant, particularly of the RNR R2 tyrosyl radical and could potentially generate uncontrolled ROS formation (Figure 2). The onset of these molecular processes is expected to result in cell death, although we cannot discount that increased cellular uptake of DefNEtTrp, in comparison to Trp, NEtTrp, and Def, is in part responsible for the observed cytotoxicity.

An additional cell viability experiment was performed to examine the effect that supplementation of Fe(III) to Jurkat cells, prior to treatment with a cytotoxic dose of DefNEtTrp, would have. Jurkat cells were supplemented with 30 μM Fe(III)-saturated serum transferrin (Fe_2_-sTf-(CO_3_)_2_) 2 h before addition of 2 μM DefNEtTrp.. Additional experiments were run in which cells were treated with DefNEtTrp alone, cells were pretreated with Fe_2_-STf-(CO_3_)_2_ without the presence of DefNEtTrp, and cells were treated with no treatment at all. Control samples were prepared containing media without cells in the presence and absence of Fe_2_-STf-(CO_3_)_2_. After 68 h of setting up these samples following the two h of Fe presupplementation, 60 μL of media was removed from all of them to measure the Fe content in them by the ferrozine assay. The samples containing cells were then quickly washed with 1× PBS buffer and then restored to working volumes of 100 μL with fresh media. After an additional 4 h, the MTT viability assay was performed. The cells that were presupplemented with Fe(III) exhibited a significant 115-fold increase in viability relative to the nonsupplemented group (Figure S20). Note that the amount of Fe presupplemented to the Jurkat cells in the absence of DNT was somewhat toxic, as it caused a 20% decline in cell viability. To correlate the changes in cell viability with Fe uptake in the cells, the Fe levels in the media were measured in all of the samples (Figure S21). In the absence of Fe_2_-sTf-(CO_3_)_2_ supplementation, there was 3.9 ± 0.6 μM Fe present in the media. In the presence of Fe_2_-sTf-(CO_3_)_2_, there was 64.5 ± 2.6 μM Fe in the medium, which matches the amount added to the medium accounting for the background Fe in the media. The Fe level in the media dropped to 61.5 ± 1.7 μM for the cells treated with Fe_2_-sTf-(CO_3_)_2_ alone, which, although not found to be statistically different from the samples without cells (*p* value = 0.1), might be the result of Fe uptake into the cells pushing the internal Fe amount slightly above the toxicity threshold. In the cells treated with the combination of Fe_2_-sTf-(CO_3_)_2_ and DefNEtTrp, the Fe level in the medium was found to be statistically lower (57.1 ± 2.8 μM; *p*-value = 0.008), which is in line with the expectation that there would be an increase in the amount of Fe within the cells and that the supplemented Fe would offset the perturbation of the intracellular LIP by DefNEtTrp.

In an analogous experiment, the cytotoxicity of Fe_3_(DefNEtTrp)_2_ was also evaluated. At 10 μM (a concentration determined by ICP-OES due to the low aqueous solubility of the compound), the compound modestly decreased cell viability by 14% (Figure S22). The compound exhibits inferior cytotoxic behavior as compared to the metal-free dual chelator possibly from reactivity inertness from the high Fe affinity coordination in this particular speciation. Such speciation may not occur in the context of how the ligand interacts with the LIP due to rapid metal turnover (Figure 2). Previous cell viability studies with Fe(III)-bound Def and Fe(III)-bound Trp demonstrated that the metal compounds were significantly less cytotoxic than the metal-free ligand.^20,27^

### Cancer Selectivity of DefNEtTrp

Given its broad-spectrum and potent cytotoxicity against different cancer cells, DefNEtTrp was evaluated against two human noncancer cell lines: MRC-5 lung cells (Figure S23) and red blood cells (Table S4). The compound demonstrated no antiproliferative behavior against MRC-5 in the concentration range examined (maximum concentration of 100 μM). Up to a concentration of 50.3 μM, the compound induced minor (∼5%) hemolysis (lysis of red blood cells) comparable to the behavior of its constituent groups, triapine, NEtTrp, and Def. These concentrations are well above the GI_50_/IC_50_ and TGI values of DefNEtTrp against all of the cancer cells examined in this work, indicative of the compound being highly selective for cancer cells.

### Cell Death Pathways Induced by DefNEtTrp

**Figure 3:**
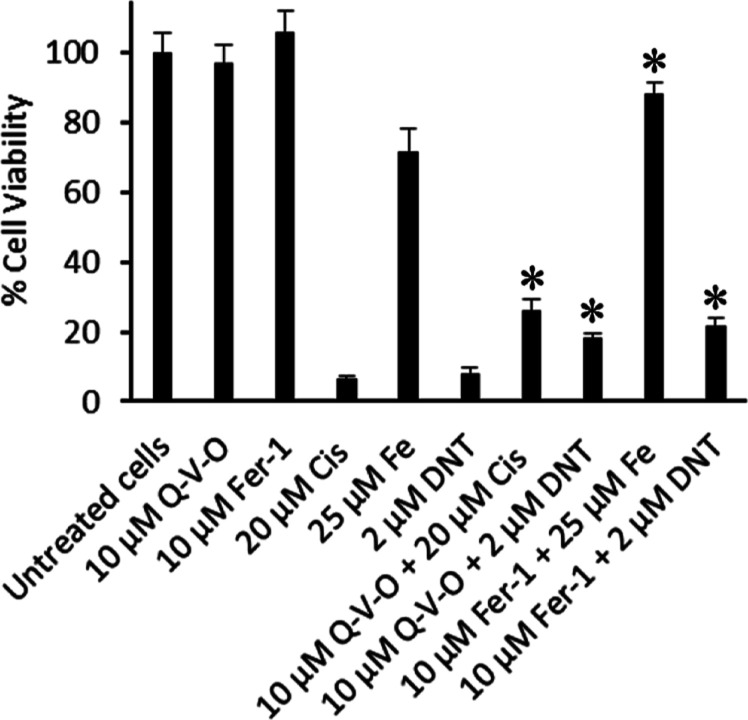
Observed changes in the % cell viability of Jurkat cells treated with caspase-dependent apoptosis and ferroptosis inhibitors. The cells were pretreated with 10 μM Q-VD-OPh (Q–V–O) or 10 μM ferrostatin-1 (Fer-1) and then treated for 72 h with 2 μM DefNetTrp (DNT), 20 μM cisplatin (Cis), 25 μM Fe(citrate)_2_ (Fe), or media alone. * *p* value <0.01 vs the corresponding treatment without the inhibitor.

Previous studies have demonstrated that the Def ligand can induce apoptotic cell death,^20^ whereas the Trp ligand can induce both apoptosis and ferroptosis.^28^ With this in mind, the apoptotic and ferroptotic capability of DefNEtTrp was examined in Jurkat cells using the apoptosis inhibitor Q-VD-OPh and the ferroptosis inhibitor ferrostatin-1 (Fer-1), followed by apoptosis and ferroptosis activity assays. Jurkat cells pretreated with Q-VD-OPh (10 μM) experienced a 2.3-fold increase in viability against treatment with 2 μM DefNEtTrp, which suggests that the dual chelator can induce caspase-dependent apoptosis (Figure 3). This result compares favorably with the finding that the apoptosis-inducing cisplatin (20 μM) experiences less antiproliferative activity against Jurkat cells when pretreated with the inhibitor. A caspase-3 activity assay was performed using a detection kit (Abcam, #ab252897), which measures the activity of the apoptotic factor caspase-3 using the synthetic substrate DEVD-AFC (7-amino-4-trifluoromethylcoumarin). The cleavage of the substrate is measured by fluorescence (*E*_x_/*E*_m_ = 400/505 nm). Jurkat cells were reacted with media alone, 20 μM cisplatin, or 2 μM DefNEtTrp for 72 h. It was observed that both DefNEtTrp (2.25-fold) and cisplatin produce a higher substrate cleavage compared to the media alone control (Figure S24). Work with the ferroptosis inhibitor, Fer-1, also revealed that DefNEtTrp triggers ferroptosis cell death given the 2.6-fold increase in the cell viability of Jurkat cells pretreated with the inhibitor (10 μM) in the presence of 2 μM DefNEtTrp (Figure 3). This behavior was supported by the positive control Fe(citrate)_2_ at a relatively high concentration (25 μM).^42^ To confirm the induction of ferroptosis, an activity assay was performed using the lipid peroxidation assay kit (Abcam, #ab118970), which measures the concentration of malondialdehyde (MDA), an end product of fatty acid peroxidation. Jurkat cells were reacted with media alone, 25 μM Fe(citrate)_2_, or 2 μM DefNEtTrp for 72 h. The fluorescence of the MDA-thiobarbituric acid adduct formation at *E*_x_/*E*_m_ = 532/553 nm was measured to obtain the relative formation of lipid peroxidation. It was observed that both DefNEtTrp (21.1 fold) and Fe(citrate)_2_ produce a higher adduct formation compared to the media alone control (Figure S25). A cotreatment inhibitor experiment was performed with equimolar Q-VD-OPh and Fer-1, which resulted in a 3.8-fold increase in the viability of Jurkat cells against treatment with 2 μM DefNEtTrp (Figure S26). The triggering of apoptosis and ferroptosis could be anticipated given the proposed redox cycling of DefNEtTrp and its potential to inhibit RNR R2.

## Conclusions

This work establishes the rich anticancer potential of dual chelator ligand DefNEtTrp. Designed to operate by reaction with the LIP of the intracellular environment, it retains the Fe(II) and Fe(III) binding capacity of its constituent chelating moieties and readily reacts with labile Fe(III) without scavenging the metal ion from sTf. The ligand should also readily bind with labile Fe(II) as indicated by the cyclic voltammetry studies, but a formal study of Fe(II) complexation is warranted. Though DefNEtTrp lacks the oral druglikeness of its constituent chelators, DefNEtTrp exhibits a highly attractive potent antiproliferative broad-spectrum cytotoxic profile specific to cancer cells.

A comparative study of the cytotoxicity of the dual chelator ligand versus that of the unconjugated chelators in Jurkat cells demonstrates that not only is the antiproliferative behavior of DefNEtTrp superior, but also its conjugation appears to facilitate a synergism that may be owed to the redox activity of Fe coordination to both chelator moieties (Figure 2). Fe(III) binding at the Trp moiety, at minimum, exhibits a reduction potential well within the biological window to be an effective Fe(II) reductant of the RNR R2 tyrosyl radical and thus inactivate the enzyme and also redox cycle. Fe(III) monoDef species that may form intracellularly could also redox cycle.^21^ This redox cycling potential warrants further study. A valuable parameter to evaluate is the cellular uptake of DefNEtTrp to directly compare its uptake with that of the unconjugated chelators. That the cell death capability of DefNEtTrp depends on its Fe-binding ability is further supported by the observation that cells presupplemented with Fe display higher resistance toward the cytotoxicity of the dual chelator. The Fe-dependent processes induced by DefNEtTrp result in the triggering of both apoptotic and ferroptotic cell death pathways. In total, a simple conjugation of two chelator moieties elucidates a new direction in the use of coordination chemistry for the clever manipulation of labile cellular physiological metals for the therapeutic effect.

## Methods

### Chemicals and Materials

*N*-Boc-ethylenediamine (1), methyl iodide, 3-aminopicolinaldehyde, (2-formyl-pyridin-3-yl)-carbamic acid *tert*-butyl ester, hydroxybenzotriazole (HOBt), 200 Proof ethanol, ethyl acetate (98%), anhydrous dimethylformamide (DMF) (99.8%), hydrochloric acid (HCl) (12 M), sodium hydroxide (NaOH) pellets, molecular sieves, sodium bicarbonate, sodium sulfate, iron(III) chloride (FeCl_3_), hydroxylamine hydrochloride, trichloroacetic acid, and tetrabutylammonium hexafluorophosphate were purchased from Sigma. Carbon disulfide and hydrazine hydrate were purchased from Alfa Aesar. 1-Ethyl-3-(3-(dimethylamino)propyl)carbodiimide was purchased from Fisher Scientific. Ethylene dichloride and deuterated dimethyl sulfoxide (DMSO-*d*_6_) were purchased from Merck Millipore. Triapine (Trp) was purchased from MedChemExpress LLC. Deferasirox (Def) was synthesized by modifying a previously reported method.^33^ Tricitratoiron(III) ([Fe(citrate)2]5–) was prepared in situ by reacting equimolar amounts of iron(III) citrate [Fe(C_6_H_5_O_7_)] (Sigma), and trisodium citrate (Na_3_ citrate) (Thermo Scientific) in solution and adjusting the pH to 7.4.^19^ High-purity Fe standard was obtained from PerkinElmer and used for ICP-OES and ferrozine quantitative assays. ICP-OES-quality HNO_3_ was obtained from a BDH Aristar. Ferrozine was obtained from Acros Organics. Jurkat cell clone E6–1 was obtained from ATCC (ATCC TIB-152) authenticated with a certificate of analysis. Roswell Park Memorial Institute (RPMI) 1640 medium (Corning, CellGro, R8758) containing l-glutamine was purchased from Sigma and supplemented with 10% fetal bovine serum (FBS; HyClone) and 1% of antibiotic solution prepared with 11 mg/mL streptomycin and 7 mg/mL penicillin purchased from Sigma. MRC-5 human lung cells were obtained from ATCC (CCL-171) authenticated with a certificate of analysis. The cell line was cultured in phenol red DMEM (Sigma, D6429) containing 1% glutamine, 4.5 g/mL glucose, and sodium pyruvate. The medium was supplemented with 10% FBS (HyClone) and 1% penicillin–streptomycin at 37 °C in a humidified atmosphere of 5% CO2 (v/v). Phenol red-free DMEM (CellGro REF 17-205-CV) was used during cell viability assay studies. 3-(4,5-Dimethylthiazol-2-yl)-2,5-diphenyltetrazolium bromide (MTT) was purchased from EMD Biosciences Inc. Tris was purchased from Amresco. Dodecyl sulfate sodium salt (SDS, electrophoresis, 98% pure) was obtained from Acros Organics. Trypan blue solution (0.14%) was purchased from Sigma. Phosphate buffer saline (10× and 1× PBS) was prepared in the lab (pH = 7.2). A Corning Costar 96-Well, Cell Culture-Treated, Flat-Bottom microplate was used to perform the cell viability assays. Ferrostatin-1, Q-VD-OPh, and cisplatin (98%) were purchased from MedChemExpress. Human apo-serum transferrin (apo-sTf) was obtained from Sigma (T2036). The purity of the protein was confirmed by SDS-PAGE. A Microsep 3K MWCO spin dialyzer was obtained from Pall Corp. This dialyzer was used to buffer wash the apo-transferrin to rid of any salts from commercial isolation of the protein before experimental use. Fe(III)-saturated serum transferrin (Fe_2_-sTf-(CO_3_)_2_) was prepared by reacting apo-sTf with four equivalents of Fe(NTA)_2_ in 20 mM Tris buffer (pH 7.4, 0.1 M NaCl, 27 mM NaHCO_3_). The protein was then washed by spin dialysis to rid the unreacted protein. The stoichiometric ratio of 1:2 Fe:sTf was confirmed by measuring the Fe concentration by the ferrozine assay and the protein concentration by the Bradford assay. The successful preparation of Fe_2_-STf-(CO_3_)_2_ was confirmed by measuring the absorbance at 465 nm for the Fe(III) LMCT band (ε = 5200 M^–1^ cm^–1^). All other chemicals and solvents were of high purity and used as received. All aqueous solutions were prepared with autoclaved (121 °C and 18 psi) high-quality nanopure water (18.2 MΩ^•^cm resistivity at 25 °C), PURELAB flex system (ELGA LabWater Corp.). Ar and CO2 USP gases were supplied by Messer Gas.

### Instrumentation

A Nicolet iS50 FTIR spectrometer (Thermo Fisher Scientific) was used to collect the FTIR absorbance spectra. Proton (^1^H) and carbon (^13^C) NMR spectra were recorded on a Bruker Avance 500 MHz NMR spectrometer, using DMSO-*d*_6_ as the solvent. Chemical shifts were recorded in units of parts per million (ppm, δ) relative to the peak of the internal solvent standard. 1H NMR data are reported as follows: chemical shift (ppm), multiplicity (singlet (s), doublet (d), triplet (t), quartet (q), multiplet (m), coupling constant in Hz, and integration). ^13^C NMR data are reported by chemical shift (ppm). Mass spectra were obtained using a Waters Xevo G-25 QTof in positive ion mode, analyzer resolution mode, capillary (kV): 3.0000, sampling cone: 40.0000, source temperature (°C): 100, source offset: 60, desolvation temperature (°C): 350, cone gas flow (L/Hr): 10.0, and desolvation gas flow (L/Hr): 600.0. Mass spectra were also obtained by using an AB SCIEX 4800 MALDI-TOF/TOF mass spectrometer. The matrix for all samples examined by MALDI-TOF MS was composed of 5 mg/mL α-cyano-4-hydroxycinnamic acid and was prepared in a solvent composed of acetonitrile and water in a 1:1 (v/v) ratio plus trifluoroacetic acid at 0.1% (v/v). 1 μL of matrix was placed on the MALDI wells and allowed to crystallize. Then, 1 μL of sample solutions was placed on top of the crystallized matrix and allowed to crystallize. Excel was used to tabulate the data, and Isopro3.1 was used to obtain the theoretical spectra of the species identified. The theoretical and experimental intensities were normalized to a maximum signal intensity of 100. The cyclic voltammograms were obtained using an SP-240 potentiostat (Biologic Science Instrument). The UV–vis spectra were obtained by using a Cary 300 UV–vis spectrophotometer (Agilent Technologies). All pH measurements were performed using a Thermo Fisher Scientific Orion Star A211 and a StrataChemometer Orion 9157BNMD electrode. The electrode was calibrated in units of mV, using standard buffer solutions at pH = 4.01, 7.00, and 10.01. X-ray diffraction analysis was carried out using a Rigaku XtalLAB SuperNOVA single microfocus Cu-Kα radiation (λ = 1.5417 Å) source equipped with a HyPix3000 X-ray detector in transmission mode operating at 50 kV and 1 mA within the CrystallizPRO software ver. 1. 171.39.43c. ICP-OES data were collected by using an Optima 8000 (PerkinElmer) system. SEM-EDS data were collected using a JSM-IT500HR instrument (JEOL Ltd.) with a scan time of 0.5 s, 20 kV voltage, probe current number 76, and high vacuum mode. The EDS detector was a DRY SD30 (JEOL Ltd.). Cw-EPR spectra were collected at the University of Arizona, EPR Facility on an X-band EPR Elexsys E500 spectrometer (Bruker) equipped with an ESR900 flow cryostat (Oxford instruments). Cell viability was determined using an MTT assay. Multiwell plate absorbance was measured in an Infinite M200 PRO Tecan Microplate Reader. Cells were grown in a Revco Elite III RCO5000T-5-ABC incubator purchased from Thermo Fisher Scientific. Cell counting was performed by the trypan blue method (1:5) using a hemocytometer. The counting and culture viability monitoring were performed by using a Nikon Eclipse TS-100 microscope.

## Supporting Information Available

Methods section; ^1^H and ^13^C NMR, FTIR, and MALDI-TOF mass spectra of compounds; pH-dependent aqueous speciation model of Fe(III) in interaction with Def and Trp from pH 2 to 8; SEM-EDS and cyclic voltammetry of Fe_3_(DefNEtTrp)_2_; NCI-60 cancer cell line viability screen results; dose–response curves for the treatment of Jurkat and MRC5 cells with compounds in single-compound or combined compound treatment; cell viability assay results in the presence of apoptosis and ferroptosis inhibitors and complementary apoptosis and ferroptosis activity screens; hemolysis assay results; crystallographic information for the X-ray study for **4**; and hemolysis studies with the compounds (PDF)
Crystallographic data of **4** (CIF)

## Supplementary Material

au4c00774_si_001.pdf

au4c00774_si_002.cif
