# Supplementary material for: DefNEtTrp: An Iron Dual Chelator Approach for Anticancer Application
Source: JACS Au. 2024 Dec 4;4(12):4799–808. doi: 10.1021/jacsau.4c00774 (PMC11672142; doi:10.1021/jacsau.4c00774)
Supplement: Supplementary file 1 — au4c00774_si_001.pdf [file au4c00774_si_001.pdf]

Supporting Information

DefNEtTrp: An Iron Dual Chelator Approach for Anticancer Application

Israel Rodríguez,[†,§] Carmen Acosta,[†,§] Christopher Nieves-Escobar,[†] Estelle Strangmark,[†] Oscar Claudio-Ares,[†] Adriana I. Vargas Figueroa,[†] Alexandra M. Soto-Millán,[†] Aixa M. Orta-Rivera,[†] Andrei V. Astashkin,[‡] and Arthur D. Tinoco*,[†]

[†] Department of Chemistry, University of Puerto Rico, Río Piedras Campus, Río Piedras, Puerto Rico 00931, United States.

[‡] Department of Chemistry and Biochemistry, The University of Arizona, Tucson, Arizona 85721-0041, United States.

§ Equal contribution.

*Email: arthur.tinoco@upr.edu

Table of Contents

**1. Methods**	3-11
**2. Supporting Figures**	12-29
Fig. S1. Ellipsoid plot of NEtTrp·2HCl (4).	12
Fig. S2. ^{1}H NMR spectrum of methyl-N-(2-tert-butoxycarbonylaminoethyl)dithiocarbonate (2).	12
Fig. S3. ^{1}H NMR spectrum of t-butyl(2-hydrazinecarbothioamide ethyl)carbamate (3).	13
Fig. S4. ^{1}H NMR spectrum of NEtTrp·2HCl (4).	14
Fig. S5. ESI-MS (positive ion mode) of DefNEtTrp (6): *m/z* 594.23, $\{(H^{+})[C_{30}H_{27}N_9O_3S]\}^{+}$ (H^{+} adduct of DefNEtTrp).	15
Fig. S6. ^{1}H NMR spectrum of DefNEtTrp (6).	16
Fig. S7. ^{13}C NMR spectrum of DefNEtTrp (6).	17
Fig. S8. ESI-MS (positive ion mode) of $Fe(DefNetTrp)_2$ (7): *m/z* 1240.41, $\{2H^{+} + [C_{60}H_{50}N_{18}O_6S_2Fe(III)]\}^{+}$.	18
Fig. S9. UV-vis spectra for Deferasirox (black line) and $Fe(Deferasirox)_2$ (dotted line) collected at pH 7.4 (50:50 (v/v) DMSO:H_2O solution (0.1 M Tris, 0.1 M NaCl).	18
Fig. S10. The pH dependent aqueous speciation model for the interaction of 50 μM Fe(III) and fully deprotonated Def^{3-} and Trp^{-} at 100 μM each.	19
Fig. S11. Formation studies of $Fe_3(DefNEtTrp)_2$ (8) from the reaction of $Fe(DefNEtTrp)_2$ (7) and varying concentrations of Fe(III) up to five mole equivalence.	20
Fig. S12. Characterization of the $Fe_3(DefNEtTrp)_2$ (8) compound. Scanning electron microscopy image of the compound (a). Energy-dispersive X-ray spectrum displaying the elements present in the compound. Trace amounts of Al and Si are observed that are background contaminants and are not part of the compound composition.	21

Fig. S13. FT-IR spectra of DefNEtTrp (6), $Fe(DefNEtTrp)_2$ (7), and $Fe_3(DefNEtTrp)_2$ (8).	21
Fig. S14. UV-vis spectra for Triapine (dotted line), Fe(Trp) (pH 2; dashed line) and $Fe(Triapine)_2$ (pH 8, line).	22
Fig. S15. The EPR spectra for Fe(Trp) (pH 2, line) and $Fe(Triapine)_2$ (pH 8, dotted line).	22
Fig. S16. MALDI-TOF MS (positive ion mode): *m/z* 1240.41, $\{(H^+)_2[C_{60}H_{50}N_{18}O_6S_2Fe]\}^+$ ($Fe(DefNetTrp)_2$) (7); *m/z* 1293.32, $\{C_{60}H_{49}N_{18}O_6S_2Fe_2\}^+$ (($Fe_2(DefNetTrp)_2$); *m/z* 1348.10, $\{C_{60}H_{48}N_{18}O_6S_2Fe_3\}^+$ ($Fe_3(DefNetTrp)_2$) (8).	23
Fig. S17. Cyclic voltammogram of 6 mM $Fe(Def)_2$.	24
Fig. S18. Dose response curves of DefNEtTrp (6) against the nine different panels of cancer cell lines in the NCI-60 five dose screen.	25
Fig. S19. Dose response curves for DefNEtTrp (6) (green), NEtTrp (4) (blue), Def (5) (orange), Trp (purple), 1:1 combination of Def and NEtTrp (black), 1:1 combination Def and Trp,(pink) against the Jurkat Leukemia cell line for 72 h.	26
Fig. S20. Observed changes in the % cell viability of Jurkat cells presupplemented with 30 μM Fe(III)-saturated serum transferrin (Fe_2-STf-$(CO_3)_2$) (pH 7.4) for two hours and then treated for 72 h with 2 μM DefNEtTrp (DNT) **(6)** or media alone.	26
Fig. S21. Observed changes in the Fe levels in the Jurkat cell media with and without 30 μM Fe(III)-saturated serum transferrin (Fe-sTf) (pH 7.4) and with and without 2 μM DefNEtTrp (DNT) (6) or media alone. Cells were treated with Fe-sTf for two hours prior to the addition of DNT.	27
Fig. S22. Dose response curve for $Fe_3(DefNEtTrp)_2$ (8) against Jurkat cells for 72 h.	27
Fig. S23. Dose response curve for DefNEtTrp (6) against the MRC-5 noncancer lung cell line for 72 h.	28
Fig. S24. Caspase-3 apoptosis activity assay in Jurkat cells with media alone (untreated), 2 μM cisplatin, or 2 μM DefNetTrp (DNT) (6).	28
Fig. S25. Ferroptosis activity assay following the formation of MDA after 72 h in Jurkat cells with media alone (untreated), 25 μM $Fe(citrate)_2$ (Fe), or 2 μM DefNetTrp (DNT) (6).	29
Fig. S26. Observed changes in the % cell viability of Jurkat cells treated with 10 μM Ferrastatin-1/Q-VD-OPh (Fer-1/Q-V-O) in combination and then treated for 72 h with 2 μM DefNetTrp (DNT) (6), or media alone. *p-value < 0.01 vs the corresponding non-treated Fer-1/Q-V-O group.	29
**3. Supporting Tables**	30-32
Table S1. Crystal and structure refinement for NEtTrp·2HCl (4).	30
Table S2. A summary of the NCI-60 cancer cell line viability screen of DefNEtTrp (6) at the five-dose level for 48 h.	31
Table S3. The calculated average 50% growth inhibition (GI_{50}) of Triapine in μM compound concentration.	31
Table S4. Evaluation of the hemolytic activity of the compounds Trp, NEtTrp (4), Def (5), and DefNEtTrp (6). Positive control: melittin. D_{max} is the maximal percentage of hemolysis measured.	32
**4. References**	33

1. Methods

Fe quantification by the ferrozine assay and ICP-OES. Depending on the concentration of Fe and volume of a given sample, either the ferrozine assay[1] (≥100 μL of 2.5 to 75 μM levels) or ICP-OES (few μL of sub μM levels) were used to quantify Fe content. For the ferrozine assay, high purity Fe standard was diluted in 0.01 M HCl(aq) to prepare appropriate stock solutions for the standard series (0.5 to 15 μM) using a Tecan Plate reader approach established in this work. The sample solutions (n=4; prepared at an estimated 7.5 μM concentration at 50 μL) and the external Fe(III) standard solutions were prepared with an exact background match to avoid matrix effects. These solutions consisted of 0.029 M hydroxylamine hydrochloride (to reduce the metal to Fe(II)), 0.21 mM ferrozine, and 1.08 M sodium acetate buffer (pH 5.2) and were left to equilibrate for one hour at room temp. The solution absorbances at 562 nm (ε = 27,000 $M^{-1}cm^{-1}$) and at 800 nm (as a measure of the baseline) were collected using the Tecan plate reader. The absorbance at 800 nm was subtracted from that at 562 nm. The corrected absorbance was plotted versus Fe(II) concentration. The concentration of Fe in the samples was determined from the line of best fit for the calibration curve. For samples containing protein, a protein digestion was necessary. The samples were mixed with an equal volume of 30% (w/v) TCA (aq) and then were boiled for five minutes. They were then sonicated for 1 h. Afterwards, the supernatant was collected following centrifugation at 10 k rpm. The supernatant was then used to carry forward the ferrozine assay. For the assays involving protein samples, the same amount of TCA was added to standards to account for matrix effects.

For the ICP-OES analysis, Fe standards from 2.5-100 ppb were prepared in 2% HNO_3 supplemented with the sample solvent to obtain an exact background and volume match as the sample. The sample (10 μL) was diluted to a final volume of 15 mL in 2% HNO_3 (v/v) to try to obtain a concentration in the middle of the concentration range of the standards. All ICP-OES data acquisition was run in axial mode for optimal sensitivity. A self-aspirating nebulizer (Meinhard) with a cyclonic type spray chamber was used. The Ar flow rate and the auxiliary gas flow rate were fixed at 15.0 L/min and 0.2 L/min throughout the experiment, respectively. In this work, the rf power was fixed at 1300 W. Sample flow rate was maintained at 1.50 mL/min. Samples were analyzed for Fe at 238.204 nm. All the data was processed with the WinLab32 software version 5.4 (Perkin-Elmer). The solution emission intensity versus Fe concentration was plotted after correcting for the emission of the blank. The concentration in the sample was determined from the line of best fit to the standard curve.

Synthesis of methyl-N-(2-tert-butoxycarbonyl amino ethyl)dithiocarbonate (2). N-boc-ethylenediamine **(1)** (2.0115 g, 12.23 mmol, 1 eq.) was dissolved in ethanol (70 mL) in a 250 mL round bottom flask equipped with a stir bar. Triethylamine (2.04 mL, 14.68 mmol, 1.2 eq.) was added to the flask and the solution was left to stir for five minutes. Carbon disulfide (887 μL, 14.68 mmol, 1.2 eq.) was added to the flask and left to stir for 90 minutes. Methyl iodide (0.914 mL, 14.86 mmol, 1.2 eq.) was then added to the flask and left to stir overnight. Ethyl acetate (30 mL) was added to the flask, resulting in the formation of white crystals. The supernatant was separated from the precipitate through two rounds of centrifugation (4.4k rpm, 45 min). Ethyl acetate was used to wash the pellets between the two rounds of centrifugation. The supernatant was concentrated using a rotary evaporator, producing a pale yellow, viscous residue. The residue was washed with 1M HCl (20 mL), saturated aqueous sodium bicarbonate (20 mL), and water (20 mL). The aqueous layer was removed between each wash. The organic layer was concentrated using the rotary evaporator and high vacuum. Methyl-N-(2-tert-butoxycarbonyl amino ethyl)dithiocarbonate **(2)** was obtained as a pale yellow, viscous oil (2.5 grams, 82% yield). In this work, % yield was determined after purification based on the calculation: experimental mass/theoretical mass x 100%. ^{1}H NMR (500 MHz, DMSO-d_6): δ = 9.80 (s, 1H), 6.90 (d, J = 5.3, 1H), 3.61 (q, J = 5.9, 2H), 3.15 (q, J = 5.9, 2H), 2.52 (q, 3H), 1.39 (s, 9H).

Synthesis of tert-butyl (2-hydrazinecarbothioamide ethyl)carbamate (3). Methyl-N-(2-tert-butoxycarbonyl amino ethyl)dithiocarbonate **(2)** (2.4 g, 9.59 mmol, 1 eq.) was dissolved in ethanol (48 mL) in a 100 mL round bottom flask. Hydrazine hydrate (0.800 mL, 16.3 mmol, 1.7 eq) was added to the flask and the solution was refluxed overnight using a vertical condenser. The solvent was removed from the flask using rotary evaporation. Ethyl acetate (25 mL) was added to the flask and the organic layer was washed with water (20 mL) three times. The organic layer was dried over anhydrous sodium sulfate and the solvent was removed using a rotovap. The sample was then concentrated overnight using a high vacuum. The thiosemicarbazide (3) was obtained as a pale, yellow viscous oil (1.7 g, 76% yield). ^{1}H NMR (500 MHz, DMSO-d_6): δ = 8.70 (s, 1H), 7.96 (s, 1H), 6.91 (t, J = 5.0, 1H), 4.45 (s, 2H), 3.50 (q, J = 5.8, 2H), 3.07 (q, J = 5.87, 2H), 1.38 (s, 9H).

Synthesis of nitrogen ethyl triapine dihydrochloride (NEtTrp·2HCl) (4). Tert-butyl (2-hydrazinecarbothioamide ethyl)carbamate (3) (1.37 g, 5.83 mmol, 1 eq.) was dissolved in ethanol (27 mL) in a 100 mL two-neck round bottom flask. 3-amino picoaldehyde (0.750 g, 5.83 mmol, 1 eq.) was added to the flask and refluxed for ten minutes. Hydrogen chloride (1.94 mL, 23.32 mmol, 4.0 eq.) was added dropwise to the stirring solution, resulting in a color change from yellow to a dark orange solution. The solution was left to stir overnight. The following day, a yellow precipitate in the orange solution was collected through two rounds of centrifugation (4.4k rpm, 20 min). Ethanol (10 mL) was used to wash the product between the two rounds of centrifugation. The supernatant was removed, and the product was concentrated under high vacuum overnight. NEt-Triapine·2HCl **(4)** was obtained as a yellow crystalline solid (1.56 g, 86% yield). MALDI-TOF (positive): m/z 239.06, $\{(H^+)[C_9H_{14}N_6S]\}^+$. ^{1}H NMR (500 MHz, DMSO-d_6): δ = 12.26 (s, 1H), 9.50 (s, 1H), 8.39 (s, 1H), 8.24 (s, 3H), 8.03 (d, J = 5.1, 1H), 7.82 (d, J = 8.6, 1H), 7.62 (dd, J_{ac} = 8.25, J_{ab} = 5.35, 1H), 7.23 (s, 2H), 3.87 (m, 2H), 3.10 (q, J = 5.63 2H). ^{13}C NMR (500 MHz, DMSO-d_6): δ = 177.68, 145.98, 133.12, 130.63, 130.45, 126.74, 126.34, 41.36, and 37.82. FT-IR (cm^{-1}) 3191, 2921, 2851, 1520, 1472, 1322, 1290, 1222, 1169, 1107, 1036, 962, 856, 792, and 702.

Synthesis of deferasirox nitrogen ethyl triapine (DefNEtTrp) (6). Def **(5)** (0.540 g, 1.45 mmol, 0.9 eq.) and hydrobenzotriazole (0.155 g, 1.01 mmol, 0.63 eq.) were dissolved in anhydrous DMF (10 mL) with a few molecular sieves in an oven-dried 100 mL round bottom flask. The flask was purged with Ar. Triethylamine (200 µL, 1.67 mmol, 1.0 eq.) was added to the flask and the mixture was stirred at 0ºC for ten minutes. A solution of EDC (0.401 g, 2.09 mmol, 1.3 eq.) in anhydrous DMF (5 mL) was added dropwise to the flask. The mixture was stirred for another thirty minutes. A suspension of 4 (0.500 g, 1.61 mmol, 1 eq.) and triethylamine (440 µL, 3.33 mmol, 2 eq.) in anhydrous DMF (5 mL) was added dropwise to the flask. The ice bath and Ar source were removed, and the solution was left to stir for 48 hours. Hydrochloric acid (10 drops, 12M) was added to the flask to promote precipitation. The precipitate was isolated through four rounds of centrifugation (4.4 k rpm, 20 min). The product was washed with water (10 mL) between each round. The solid was stored at -80ºC for a few hours before being lyophilized. DefNEtTrp **(6)** was obtained as a yellow solid (0.845 g, 68% yield). The compound was >95% pure. C,H,N elemental analysis was performed by Atlantic Microlabs (Norcross, GA). Anal. Calcd for $C_{30}H_{27}N_9O_3S{\cdot}2H_2O$ (Mr = 629.70 g/mol): C, 57.22 (57.44); H, 4.96 (5.13); N, 20.02 (19.87); Theoretical (Experimental). ESI-MS (positive): m/z 594.20, $\{(H^+) + [C_{30}H_{27}N_9O_3S]\}^+$. ^{1}H NMR (500 MHz, DMSO-d_6): δ = 11.43 (s, 1H), 10.83 (s, 1H), 10.07 (s, 1H), 8.52 (s, 1H), 8.37 (s, 1H), 8.06 (d, J = 7.1, 1H), 7.95 (d, J = 8.3, 2H), 7.85 (d, J = 3.6, 1H), 7.54 (d, J = 8.3, 4H), 7.38 (t, J = 7.7, 2H), 7.20 (d, J = 8.1, 1H), 7.09 (q, J = 4.15, 1H), 7.01 (m, 3H), 6.87 (d, J = 8.2, 1H), 6.55 (s, 2H), 3.76 (s, 2H), 3.55 (s, 2H). ^{13}C NMR (500 MHz, DMSO-d_6): δ = 177.13, 166.51, 160.28, 156.82, 155.73, 152.43, 149.79, 144.40, 140.41, 137.74, 134.44, 133.31, 132.96, 131.91, 131.55, 128.82, 127.24, 124.96, 123.66, 122.80, 120.17, 119.87, 117.54, 116.62, 114.95, 114.17, 44.83, 39.98. FT-IR (cm^{-1}): 3254, 1648, 1603, 1524, 1458, 1301, 1250, 1148, 1098, 847, 753, 656, 562, 443.

Characterization of nitrogen ethyl triapine dihydrochloride (NEtTrp·2HCl) (4) by Single Crystal X-ray Diffraction. A brown plate-like crystal of NEtTrp·2HCl **(4)** was mounted on a MiTeGen micro loop for structure elucidation. Structural elucidation was performed using a Rigaku XtalLAB SuperNova single micro-focus Cu-Kα radiation (λ = 1.5417 Å) source equipped with a HyPix3000 X-ray detector in transmission mode operating at 50 kV and 1 mA within the CrystAlisPRO software ver. 1.171.39.43c. An Oxford Cryosystems Cryostream 800 cooler controlled the temperature at 293 K. The crystal structure was solved by Intrinsic Phasing using the program ShelXT and refined by full-matrix least squares on F2 using ShelXL within the Olex2 (v1.2-ac3) software. All non-hydrogen atoms were anisotropically refined. All the hydrogen atoms were placed in their calculated positions and then refined using the riding model. Isotropic displacement parameters for these atoms were set to 1.2 times Ueq of the parent atom. A summary of the crystal data, structure solution and refinement are included in the supporting materials (Figure S1 and Table S1). CCDC 2216216 contains the supplementary crystallographic data for this paper. This data can be obtained free of charge via www.ccdc.cam.ac.uk/data_request/cif, or by emailing data_request@ccdc.cam.ac.uk, or by contacting The Cambridge Crystallographic Data Centre, 12 Union Road, Cambridge CB21EZ, UK: fax: + 44 1223 336033

Partition coefficient (log $D_{7.4}$) measurements. Partition coefficient (log $D_{7.4}$) measurements were performed adapting a literature protocol for the shake-flask procedure.[2] Stock solutions of 5 mM deferasirox **(5)**, triapine, and DefNEtTrp **(6)** were prepared in DMSO. Solutions of 5, 10, 15, 20, 30, 40, and 50 µM of the compounds in 1-octanol with 5% DMSO (v/v) and in 1X PBS (pH 7.4) with 5% DMSO (v/v) were prepared and scanned with a UV-vis spectrophotometer. The absorbance maximum was plotted versus the corresponding concentrations to produce calibration curves. The calibration curves for Def **(5)**, Trp, and DefNEtTrp **(6)** were obtained at 304 nm, 374 nm, and 378 nm in 1-octanol (5% DMSO; v/v) with extinction coefficients of 16,200 $M^{-1}cm^{-1}$, 23,300 $M^{-1}cm^{-1}$, and 28,000 $M^{-1}cm^{-1}$, respectively. The extinction coefficients in the aqueous solution at pH 7.4 (5% DMSO; v/v) were 11,400 $M^{-1}cm^{-1}$, 17,900 $M^{-1}cm^{-1}$, and 13,200 $M^{-1}cm^{-1}$, respectively. Solutions of 25 µM of all three compounds in 50:50 volume mixture of 1-octanol with 5% DMSO (v/v) and of 1X PBS (pH 7.4) with 5% DMSO (v/v) were prepared and left shaking over 3 days to equilibrate. The octanol layer was carefully extracted, and its absorbance was measured. Using the calibration curves, the concentration of the compounds in the octanol and aqueous layers were obtained. The log $D_{7.4}$ was calculated by obtaining the logarithm of the concentration of the compound in octanol divided by the concentration in the aqueous layer using the equation:

$$\log D_{7.4} = \log([\text{Compound Octanol}/\text{Compound Aqueous layer}])$$

The log $D_{7.4}$ measurements for each compound were done in triplicate.

pH dependent speciation model of Fe(III) interaction with Def (5) and Trp. A pH-dependent speciation model for the reaction of 50 µM Fe(III) with 100 µM Def **(5)** and 100 µM Trp was prepared by using the Species program developed by L.D. Pettit (Academic Software; https://www.acadsoft.co.uk/aq_solutions.htm). To generate this model, the pH-dependent formation constants for Fe(III) Def and Fe(III) Trp species,[3, 4] the pKa values of the Def **(5)** and Trp molecules,[25, 28] the relevant Fe(III) hydrolysis constants,[5] and the pKw of water were used.

Synthesis of Fe(DefNEtTrp)$_2$ (7). DefNEtTrp **(6)** (251 mg, 0.39 mmol, 1 eq.) was dissolved in 80 mL of methanol with mild heating, producing a dark yellow solution. Iron(III) dicitrate (Fe(Citrate)$_2$) was prepared in situ from the 1:1 reaction of trisodium citrate with ferric monocitrate in water. A total of 40 mL of the Fe(Citrate)$_2$ was added dropwise (0.51 mmol, 1.3 eq.) to the DefNetTrp **(6)** solution. A dark orange color solution formed. The pH of the reaction solution was adjusted to 7.0 with 1 M NaOH (aq). The solution was rotovapped to dryness. The crude product (dark brown) was suspended in water to dissolve unreacted Fe(Citrate)$_2$ and transferred into a 50 mL falcon tube. The suspension was centrifuged at 10 k rpm for 20

minutes to discard the supernatant. The pellet was washed with three cycles of water. The brown solid was isolated and lyophilized for 48 hours (195.2 mg, 73.7% yield). The compound was >95% pure. ESI-MS (positive): m/z 1240.28, {$2H^+$ + [$FeC_{60}H_{50}N_{18}O_6S_2$]}$^+$. C,H,N elemental analysis was performed by Atlantic Microlabs (Norcross, GA). Anal. Calcd for Na[Fe($C_{60}H_{50}N_{18}O_6S_2$)] · 2 H_2O · 2 CH_3OH (Mr = 1362.26 g/mol): C, 54.67 (54.68); H, 4.59 (4.9); N, 18.51 (18.57); Theoretical (Experimental). FT-IR (cm^{-1}) 3254, 1648, 1603, 1524, 1458, 1301, 1250, 1148, 1098, 847, 753, 656, 562, 443. UV Vis absorbance at 457 nm (ε = 3,600 $M^{-1}cm^{-1}$) and at 515 nm (ε = 1,800 $M^{-1}cm^{-1}$) in 50:50 (v/v) DMSO:H_2O solution (0.1 M Tris, 0.1 M NaCl, pH 7.4). The EPR spectrum (frozen 30% H_2O in DMSO (v/v), 77 K) shows a high spin Fe(III) (S = 5/2) with general rhombic symmetry (E/D ≈ 1/3) with a major line at g ≈ 4.3 and a weak feature at g ≈ 9.

Synthesis of Fe_3(DefNEtTrp)$_2$ (8). DefNEtTrp **(6)** (0.1112 g, 0.177 mmol) was dissolved in 6 mL of DMSO producing a dark yellow solution. $FeCl_3$ was dissolved in 3 mL of 1 mM HCl with stirring and moderate heating. The concentration of this solution was determined to be 391 mM by the ferrozine assay. To the DefNEtTrp **(6)** solution was added 1.5 mL of the 391 mM $FeCl_3$ solution (0.587 mmol) dropwise. The solution turned almost black in color. To it was added 4.5 mL of H_2O to yield 50:50 DMSO:H_2O (v/v). A brown-orange suspension formed. The pH of this suspension was 2.87. The pH was carefully adjusted with aliquots of 5 M NaOH until a pH of 7 to 8 to ensure that Fe(III) would bind to both the Def and Trp moieties based on published pH-dependent speciation data.[3, 4] Additional DMSO was added to maintain the mixture at 50:50 DMSO:H_2O (v/v) and the reaction was left stirring at room temp. overnight. A dark black red precipitate formed. It was isolated by centrifuging at 10 k rpm for 20 minutes in a 50 mL falcon tube. The precipitate was washed through four rounds of centrifugation with ice-cold H_2O (4.4 k rpm, 20 min). The solid was stored at -80 ºC for a few hours before being lyophilized. Fe_3(DefNEtTrp)$_2$ **(8)** was obtained as a dark black red solid (50 mg, 29.14% yield). The compound was >95% pure. C,H,N elemental analysis was performed by Atlantic Microlabs (Norcross, GA). Anal. Calcd for $Fe_3C_{65}H_{100}N_{18}O_{28.5}S_{4.5}$; [$Fe_3(C_{30}H_{24}N_9O_3S)_2(H_2O)_6$]$(OH)_3$ · 2.5 DMSO · 12 H_2O (Mr = 1,900.80 g/mol): C, 41.07 (41.29); H, 5.30 (5.29); N, 13.26 (13.29); Theoretical (Experimental). The qualitative elemental composition was determined by SEM-EDS, which confirmed the presence of C, N, O, S, and Fe elements. The analysis was performed using 1 mg of the powdered sample which was evenly distributed on an ultrathin carbon film on a Teflon stub using a stainless steel spatula. A blower was used to rid of unadhered material. The SEM image and ED spectrum were obtained using SEM at an accelerating voltage of 20 kV and a magnification of 1,500X. MALDI TOF-MS (positive ion mode): m/z 1348.10, {Fe(III)Fe(II)$_2$ ($C_{30}H_{24}N_9O_3S$)$_2$}$^+$ (the reduced Fe was produced within the instrument). FT-IR (cm^{-1}) 3352, 1653, 1635, 1600, 1557, 1517, 1505, 1473, 1454, 1437, 1315, 1254, 1148, 1104, 1014, 984, 951, 847, 756, 718, and 683. UV Vis absorbance at 512 nm (ε = 6,400 $M^{-1}cm^{-1}$) in 50:50 (v/v) DMSO:H_2O solution (0.1 M Tris, 0.1 M NaCl, pH 7.4). The EPR spectrum (frozen 30% H_2O in DMSO (v/v), 77 K) shows a high spin Fe(III) (S = 5/2) with general rhombic symmetry with a major line at g ≈ 4.3 and a weak feature at g ≈ 9. It also shows a low spin Fe(III) (S = 1/2) with a rhombic symmetry (g_z ~2.19; g_y~2.14; g_x ~2.00).

In situ preparation of Fe(Def)$_2$ (9). The synthesis of Fe(Def)$_2$ **(9)** was done as follows. Deferasirox (18.8 mg, 0.05 mmol) was dissolved in 5 mL of DMSO, producing a light pale brown solution. Iron(III) dicitrate (Fe(Citrate)$_2$) was prepared as reported above in the synthesis of **(7)**. A solution of 50 µM Fe(Def)$_2$ was then prepared following speciation studies[3] by reacting one mole equivalent of the Fe(Citrate)$_2$ stock solution with 6 mole equivalents of the Deferasirox stock solution in 50:50 (v/v) DMSO:H_2O solution (0.1 M Tris, 0.1 M NaCl). The pH of the Fe(Def)$_2$ solution was adjusted to 7.4 and left to equilibrate overnight. The pH remained at pH 7.4. The UV-vis spectrum for Fe(Deferasirox)$_2$ was determined by correcting for the excess Deferasirox used to generate it.

In situ preparation of Fe(Trp) (10) and **Fe(Trp)$_2$ (11)**. Two pH-dependent Fe(III) triapine species were prepared by following speciation studies.[4] Triapine (24.8 mg, 0.02 mmol, 1 eq.) was dissolved in 5 mL of DMSO, producing an amber solution. A stock solution of Iron(III) sulfate pentahydrate ($Fe_2(SO_4)_3 \bullet 5H_2O$) was prepared in 1M HCl (aq). A solution of Fe(III) Trp was then prepared by reacting one mole equivalent of the $Fe_2(SO_4)_3$ stock solution with 2.5 mole equivalents of the triapine stock solution in 50% water in DMSO (v/v). Half of the solution was pH adjusted to 2 and readjusted as needed at equilibrium. The other half was pH adjusted to 8 and readjusted as needed at equilibrium. Each solution was divided for UV-vis and EPR measurements. They were diluted to 50 μM Fe(III) (125 μM Trp) in 50% water in DMSO (v/v) to collect their UV-vis spectra, which were corrected for the excess Triapine. They were also diluted to 1 mM Fe(III) (2.5 mM Trp) and a final 30% water in DMSO (v/v) solvent background for EPR measurements.

EPR analysis of Fe(DefNEtTrp)$_2$ (7) and Fe$_3$(DefNEtTrp)$_2$ (8) Fe(Trp) (10), and Fe(Trp)$_2$ (11). 1mM solutions of Fe(DefNEtTrp)$_2$ **(7)**, Fe$_3$(DefNEtTrp)$_2$ **(8),** Fe(Trp) **(10),** and Fe(Trp)$_2$ **(11)** were prepared in 30% water in DMSO (v/v). The samples were then transferred to EPR tubes and frozen in liquid N_2 before EPR analysis. The EPR experiments were performed using the following conditions: microwave frequency, 9.442 GHz; microwave power, 2 mW; magnetic field modulation amplitude, 0.5 mT, temperature: 77 K for high-spin Fe(III) (S = 5/2) detection; microwave frequency, 9.443 GHz; microwave power, 200 μW; magnetic field modulation amplitude, 0.5 mT, temperature: 77 K for low-spin Fe(II) (S = 1/2) detection.

Cyclic voltammetry studies of Fe(III) DefNEtTrp species. The reduction potential of 10 mL of 6 mM Fe(DefNEtTrp)$_2$ **(7)**, Fe$_3$(DefNEtTrp)$_2$ **(8)** and Fe(Def)$_2$ in DMF were examined by cyclic voltammetry (CV). The 10 mL of 6 mM of both compounds were prepared in situ in DMF. NaOH(aq) (1 M) was added to the solutions. The mole amount of NaOH added was based on a separate solution preparation with 50:50 DMF:H_2O that would achieve a raw pH of 7.4 (not solvent corrected). The solutions contained 0.2 M NBu4PF6 (diluted from a 2M stock prepared in DMF using NBu4PF6 recrystallized from 100% distilled ethanol). Cyclic voltammograms were measured in a three-electrode cell, consisting of a 2.0 mm diameter glassy carbon working electrode (polished with a polishing kit), a platinum auxiliary electrode, and an Ag/AgCl reference electrode containing 0.1 M KCl (aq). All samples were deaerated by passing a stream of Ar through them before measurements and then maintaining a blanket of Ar over them during measurements. All measurements were performed with 4 scans at a scan rate of 100 mV/s. The final results are reported relative to the normal hydrogen electrode (NHE). Appropriate solution controls were run: blank anhydrous DMF, Def **(5)**, DefNEtTrp **(6)**, and $FeCl_3$ in anhydrous DMF.

Assessing the in situ formation of Fe$_3$(DefNEtTrp)$_2$ (8) at pH 7.4. A stock solution of 80 μM Fe(DefNEtTrp)$_2$ **(7)** was prepared in DMSO. Separate 2 mL solutions of 20 μM Fe(DefNEtTrp)$_2$ **(7)** were prepared containing varying concentrations of Fe(III) (using $Fe_2(SO_4)_3$ dissolved in 0.1 M HCl(aq) up to 200 μM Fe(III) diluted in buffer (0.22 M Tris, 0.22 M NaCl, pH 7.4). The order of addition was the Fe(DefNEtTrp)$_2$ **(7)** solution first, followed by Fe(III) addition, and finally the buffer solution to yield a 50:50 (v/v) DMSO:H_2O solution (0.1 M Tris, 0.1 M NaCl, pH 7.4). These solutions were left to equilibrate for six hours and then the UV-vis spectra were collected over the wavelength range of 300 to 600 nm at 25 °C. The spectra were deconvoluted using the software *ReactLab Equilibria.* The program calculates the molar absorptivity spectra profile distribution diagram and equilibrium constants of the relative concentrations of the pure species and product formation. The data were fit according to the reaction equation below for the formation of the Fe$_3$(DefNEtTrp)$_2$ **(8)** species.

$$\mathrm{Fe(DefNEtTrp)_2^{-}} + 2\,\mathrm{Fe(III)} \rightleftarrows \mathrm{Fe_3(DefNEtTrp)_2^{3+}} + 2\,\mathrm{H^+}$$

Assessing DefNEtTrp interaction with Fe(III)-saturated serum transferrin. The ability of DefNEtTrp **(6)** to scavenge Fe(III) from Fe(III)-saturated serum transferrin(Fe_2-sTf) was also examined. Fe_2-sTf was prepared by reacting micromolar amounts of apo-sTf with 4 mol equiv of in situ prepared $[Fe(citrate)_2]^{5-}$ in 20 mM Hepes buffer (pH 7.4) containing 0.1 M NaCl and 27 mM $NaHCO_3$. The sample was extensively dialyzed by rapid spin dialysis to remove the excess unbound Fe(III). The Fe(III) content was determined by the ferrozine colorimetric assay modified from a literature protocol[6] and by monitoring the Fe_2-sTf-$(CO_3)_2$ LMCT band (λ_{max} at 465 nm; $\varepsilon = 5200\ M^{-1}cm^{-1}$).[7] To 22.5 μM Fe_2-sTf was reacted 45 μM DefNEtTrp **(6)** (diluted in the same protein buffer while maintaining 5% DMF (v/v)) in quadruplicate at 25 °C. The reaction solutions were left to equilibrate and the LMCT absorbance was monitored after 24 h and 72 h. The reaction solutions were extensively dialyzed and then the protein concentrations were quantified by the Bradford assay. Fe quantification of bound Fe was performed with the ferrozine assay. The UV-vis spectra of the washed protein samples were collected.

National Cancer Institute Cancer Cell Line (NCI-60) Screen of DefNEtTrp (6). Details of the methodology for NCI-60 cell line screening are described at http://dtp.nci.nih.gov/branches/btb/ ivclsp.html. The cells, grown in supplemented RPMI-1640 medium, are seeded in 96 well plates at an appropriate density and incubated for 24 h. The test compounds are dissolved in DMSO and incubated with cells at a single dose of 10.0 μM for 48 h. The assay is terminated by addition of cold trichloroacetic acid, and the cells are fixed and stained with sulforhodamine B. Bound stain is solubilized, and the absorbance is read on an automated plate reader. Compounds which exhibit significant growth inhibition in the one dose screen are evaluated against a 57 cell panel at five concentration levels (0.01, 0.1, 1, 10 and 100 μM). Using the seven absorbance measurements [time zero, (Ti), control growth, (C), and test growth in the presence of drug at the five concentration levels (Tf)], the percentage growth was calculated at each of the drug concentrations levels as: [(Tf-Ti)/(C-Ti)] x 100 for concentrations for which Tf ≥ Ti and [(Tf-Ti)/Ti] x 100 for concentrations for which Tf < Ti. Three-dose response parameters (GI_{50}, TGI, and LC_{50}) were calculated in the five dose assay. Growth inhibition of 50% (GI_{50}) was calculated from 100 × [(Tf-Ti)/(C-Ti)] = 50, which was the drug concentration resulting in a 50% reduction in the net protein increase (as measured by sulforhodamine B, SRB staining) in control cells during the drug incubation. The total growth inhibition (TGI) was calculated from Tf = Ti, which was the drug concentration resulting in total growth inhibition and signified the cytostatic effect. The 50% cellular death (LC_{50}) was calculated from 100 × [(Tf-Ti)/Ti] = -50, indicating a net loss of cells following treatment which indicated the concentration of drug resulting in a 50% reduction in the measured protein at the end of the drug treatment as compared to that at the beginning. Values were calculated for each of these three parameters at the level of activity; however, if the effect did not reach the level of activity, the value of parameter was expressed as less than the minimum concentration tested, or if the effect exceeded the level of activity, the value of parameter was expressed as greater than the maximum concentration tested. Log GI_{50}, log TGI, and log LC_{50} are the logarithm molar concentrations producing 50% growth inhibition (GI_{50}), a total growth inhibition (TGI), and a 50% cellular death (LC_{50}), respectively.

Jurkat cell culturing and cell viability studies. Jurkat cells are nonadherent cells, and they were cultured in an RPMI-1640 media (supplemented with 10% FBS (v/v) and 1% antibiotic solution (v/v) of penicillin/streptomycin) in non-tissue-culture treated 25 mL flasks according to the known protocol provided by the supplier ATCC. The cell passaging was done at 70% confluency by taking 3 mL of cells and diluting to 25 mL of fresh media. The cells were incubated at 37 °C in 5% CO_2 atmosphere. Jurkat cytotoxicity of $Fe_3(DefNEtTrp)_2$ **(8)**, DefNEtTrp **(6)**, Def **(5)**, NEtTrp **(4)**, Trp, and 1:1 combinations of **(5)** with **(4)** and **(5)** with Trp was determined by the colorimetric 3-(4,5-dimethylthiazol-2-yl)-2,5-diphenyltetrazolium bromide (MTT) assay. Incubation conditions were the same in all the steps. Cells in 80-95% confluence (grown in standard circular tissue culture dishes, BD Falcon) were separately seeded into 96-well plates using phenol red free RPMI-1640 media (containing 10% FBS and 1% streptomycin/penicillin) in a volume of 50 μL at a concentration of 5.0×10^5 cells/mL.

Following this procedure, the Jurkat cells were treated immediately. Stock solutions of the compounds were first prepared in DMF at 5 mM and stored at 4°C for up to 30 days. The stock solution was diluted to specific concentrations using 1X PBS (0.2−20 μM) while DMF was maintained at 1% (v/v). Due to the more limited aqueous solubility of $Fe_3(DefNEtTrp)_2$ **(8)**, the compound was tested within the concentration range of 0.075−10 μM. A total of 50 μL of diluted solutions were added in all cell-containing wells with at least six replicates per concentration (n = 6). The final percentage (v/v) of DMF was 0.5%. The cells were also co-treated with **(5)** with **(4)** and **(5)** with Trp in a 1:1 mole equivalent ratio over the same concentration range but as a combined concentration of both compounds. Control wells in the plates consisted of one lane of cells treated with media alone including 0.5% DMF (v/v) as the measure of 100% viable cell growth in the media. Another control lane consisted of no cells with media including 0.5% DMF (v/v) as a measure of the background with no viable cells. The plates were incubated for 72 h. At 4 h before completion of the incubation time, 25 μL of MTT solution (sterile filtered 1.5 mg/mL solution, dissolved in 1X PBS buffer) was added to each well. During addition of the MTT solution, the plates were protected from light. After completion, a 50 μL portion of 14% (w/v) SDS solution (sterile filtered, dissolved in Tris buffer 1M, pH =10) was added to each well and incubated to solubilize formazan crystals after MTT cell labeling. The absorbance of each well was measured at 570 nm (absorbance of the formazan product at pH > 10.0) and 800 nm (as a background correction) using a Tecan plate reader. The absorbance of all wells was compared to the absorbance of the untreated cells with the MTT set as the 100% viable cell standard and the absorbance of the no cell control set as the 0% viable cell standard. Nonlinear regression in GraphPad Prism 8 was utilized to fit the growth curve over the various drug concentrations to determine the half-maximal inhibitory concentration (IC_{50}). All cell viability experiments, including non-Jurkat cells, were repeated twice for biological duplicates. To confirm cell death at the higher concentration dosages of the compounds and combinations, dead cells were blue stained with trypan blue and visually inspected under the microscope.

Fe(III) supplementation cell viability study. Jurkat cells were seeded into 96-well plates, as indicated in the MTT assay described above. One group of cells was supplemented with 30 μM Fe_2-STf-$(CO_3)_2$ (prepared in situ). Another group of cells was treated with media alone. After 2 h incubation, half of the cells supplemented with Fe_2-sTf-$(CO_3)_2$ were treated with 2 μM DefNEtTrp **(6)** while the other half were treated with media alone. The final percentage of DMF was maintained at 0.5 % (v/v) and final total volume was 100 μL. A control lane of samples was prepared in which the protein was added to the media without any cells. At 4 h before completion of the 72 h incubation time, the 96 well plate was centrifuged (1000 rpm, 10 mins). A total of 60 μL solution was removed from each well to be used for iron quantification. Cells were then rinsed by adding 160 μL of 1X PBS and then centrifuged again to remove 160 μL of the supernatant. This process was repeated a second time. Afterwards, 60 μL of media was added to return the volume to 100 μL. The MTT assay was performed with these samples (n = 6). A student t-test was performed to evaluate the difference in the viability of cells treated with DefNEtTrp **(6)** and supplemented with and without Fe_2-sTf-$(CO_3)_2$. To the 60 μL of the samples that were removed (prior to the MTT assay) was added 60 μL of 30% (w/v) TCA (aq). They were boiled for five minutes and then left to equilibrate at room temp. for 1 h while vigorously mixing via sonication. Afterwards, the samples were centrifuged to pellet precipitated matter and to collect the supernatant. The ferrozine assay was then performed to quantify the amount of Fe in every sample.

MRC-5 cell viability studies. A thawed stock of MRC-5 cells was washed with 1X PBS and resuspended in phenol red DMEM media (supplemented with 10% FBS and 1% penicillin-streptomycin) and then seeded in a 100 mm × 20 mm (complete, O.D. x H) petri dish and grown in a 5% (v/v) CO_2 humidified atmosphere at 37 °C. At least three passages were performed to ensure the integrity of the cells. After this point, cells were collected, thoroughly washed with 1X PBS, and then resuspended in phenol red-free DMEM (supplemented with

10% FBS, 1% penicillin-streptomycin, and 2.4 mM L-glutamine) at a 2.0 × 10^5 cells/mL concentration. A volume of 50 µL of cells was seeded into 96-well plates. Cells were incubated for 24 h. Stock solutions of DefNEtTrp **(6)** were prepared fresh in DMF at concentrations of 20,000, 10,000, 5,000, 2,000, 1000, 200, 80, and 40 µM. The solutions were diluted 100-fold in 1X PBS to obtain a second set of stock solutions with 1% DMF(v/v). The MRC-5 cells were treated with 50 µL of DefNEtTrp **(6)** compound solution from the second set of stock solutions to obtain final concentrations of 100, 50, 25, 10, 5, 1, 0.4, and 0.2 µM with 0.5% DMF (v/v). Control wells were the same type of controls as in the Jurkat cell work. Cells were incubated for 68 h and the MTT assay was performed exactly as described for the Jurkat cells.

CO-ADD hemolysis assay. Trp, NEtTrp **(4)**, Def **(5)**, and DefNEtTrp **(6)** (ranging in mass 1–2 mg) were delivered to the Community for Open Antimicrobial Drug Discovery (CO-ADD). The compounds were solubilized to a stock concentration of 10 mg/mL in DMSO. Samples were serially diluted 1:2 fold in water to final testing concentrations of 32, 16, 8, 4, 2, 1, 0.5, 0.25 µg/mL while keeping the final DMSO concentration to a maximum of 0.32% (v/v). Each sample concentration was prepared in polypropylene 384-well plates (Corning 3657) for hemolysis assays, all in two duplicate plates (n =2). All sample preparation was conducted using liquid handling robots. Human whole blood (Australian Red Cross) was washed three times with three volumes of 0.9% NaCl (w/v) and resuspended in a concentration of 0.5 × 10^8 cells per mL, determined by manual cell count in a Neubauer hemocytometer. Washed cells were added to the compound-containing plates for a final volume of 50 µL. After a 10 min shake on a plate shaker the plates were then incubated for 1 h at 37 °C. After incubation, the plates were centrifuged at 1000 g for 10 min to pellet cells and debris, 25 µL of the supernatant was then transferred to reading plates (384 well, polystyrene plated (PS), Corning CLS3680), with hemolysis determined by measuring the supernatant absorbance at 405 mm (OD405) using a Tecan M1000 Pro monochromator plate reader. HC_{10} and HC_{50} (concentration at 10% and 50% hemolysis, respectively) were calculated by curve fitting the inhibition values vs. log(concentration) using a sigmoidal dose–response function with variable fitting values for the top, bottom, and slope. The maximal percentage of hemolysis is reported as Dmax. Hemolysis samples are classified by $HC_{10} \leq 32$ µg/mL. Melittin (Sigma M2272) was used as a positive hemolytic control on each plate and exhibited HC_{10} and HC_{50} values within the expected range. Melittin was used in 8 concentrations in 2-fold serial dilutions with 50 µg/mL being the highest concentration.

Cell viability study with ferroptosis and apoptosis cell death inhibitors. Jurkat cells were seeded into 96-well plates, as indicated in the MTT assay described above. Cells were treated with media alone, 10 µM of the ferroptosis inhibitor ferrostatin-1 (Fer-1), 10 µM of the apoptosis inhibitor Q-VD-OPh (Q-V-O), or a combination of 10 µM Fer-1 and 10 µM Q-V-O. After 1 h incubation, some of the cells supplemented with the cell death inhibitors were treated with 2 µM DefNEtTrp **(6)**, a corresponding positive control, or media alone. 25 µM $[Fe(citrate)_2]^{5-}$ served as a positive control for ferroptosis induction. 20 µM cisplatin served as a positive control for apoptosis induction. The final percentage of DMF was maintained at 0.5% (v/v). The MTT assay was performed with these samples (n = 6). A student t-test was performed to evaluate the difference in the viability of cells treated with DefNEtTrp **(6)**, $[Fe(citrate)_2]^{5-}$, or cisplatin and supplemented with and without Fer-1 and supplemented with and without Q-V-O.

Cell viability study with ferroptosis and apoptosis cell kit assays. Jurkat cells were seeded into 96-well plates, as indicated in the MTT assay described above. Cells were treated with media alone, 25 µM $Fe(citrate)_2$, or 2 µM DefNEtTrp **(6)** for 72 h. The final percentage of DMF was maintained at 0.5% (v/v). A lipid peroxidation assay kit (Abcam, #ab118970) was used to measure the concentration of malondialdehyde (MDA), an end product of fatty acid peroxidation. The kit measures the fluorescence of the MDA-thiobarbituric acid adduct formation at the Ex/Em = 532/553 nm. A similar approach was taken to evaluate apoptosis. Cells were treated with media alone, 20 µM cisplatin, or 2 µM DefNEtTrp **(6)** for 72 h. A

caspase-3 detection kit (Abcam, #ab252897) was used to measure the activity of the apoptotic factor caspase-3 using the synthetic substrate DEVD-AFC (7-amino-4-trifluoromethylcoumarin), which emits strong, stable fluorometric signal after being cleaved by caspase-3 (Ex/Em = 400/505 nm). The measurement was done after 2 h reaction with the substrate. Both sets of experiments were performed in triplicate (n = 3).

2. Supporting Figures

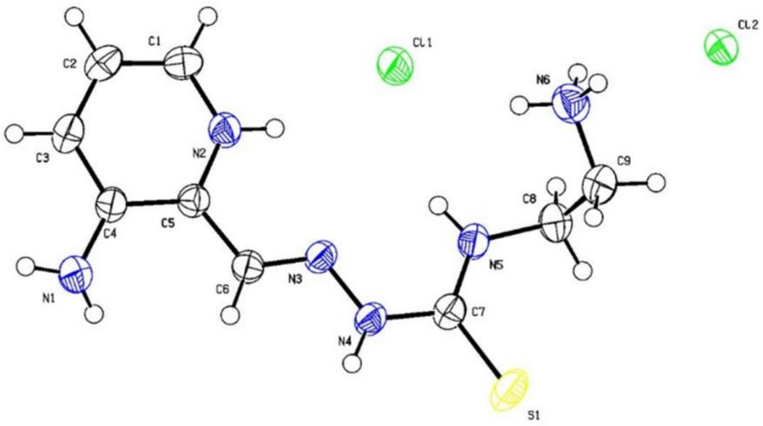

Fig. S1. Ellipsoid plot of NEtTrp·2HCl **(4)**. The thermal ellipsoids are drawn at 50% probability level. Selected bond lengths (Å) and torsion angles (deg): C6-N3 1.275(3), N3-N4 1.357(2), N4-C7 1.357(3), C7-S1 1.682(2), C7-N5 1.331(3), N5-C8 1.448(3), C8-C9 1.508(3), C9-N6 1.480(3) Å; $\Theta_{(N1\text{-}C4\text{-}C5\text{-}C6)}$ -5.8(3)°, $\Theta_{(C5\text{-}C6\text{-}N3\text{-}N4)}$ 178.3(2)°, $\Theta_{(N3\text{-}N4\text{-}C7\text{-}S1)}$ 179.7(1)°, $\Theta_{(N3\text{-}N4\text{-}C7\text{-}N5)}$ -1.0(3)°, $\Theta_{(N5\text{-}C8\text{-}C9\text{-}N6)}$ -69.5(2)°

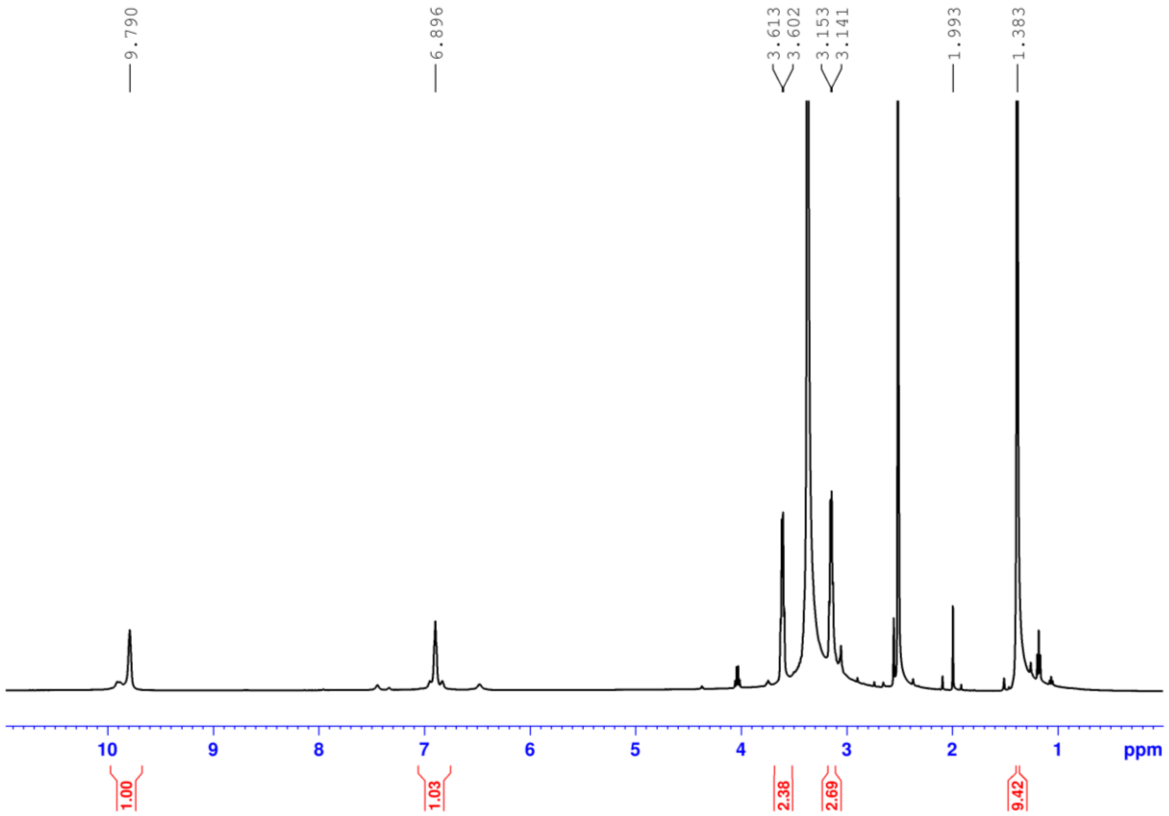

Fig. S2. ^{1}H NMR spectrum of methyl-N-(2-tert-butoxycarbonylaminoethyl)dithiocarbonate **(2)**. [Conditions: 500 MHz, DMSO-d_6].

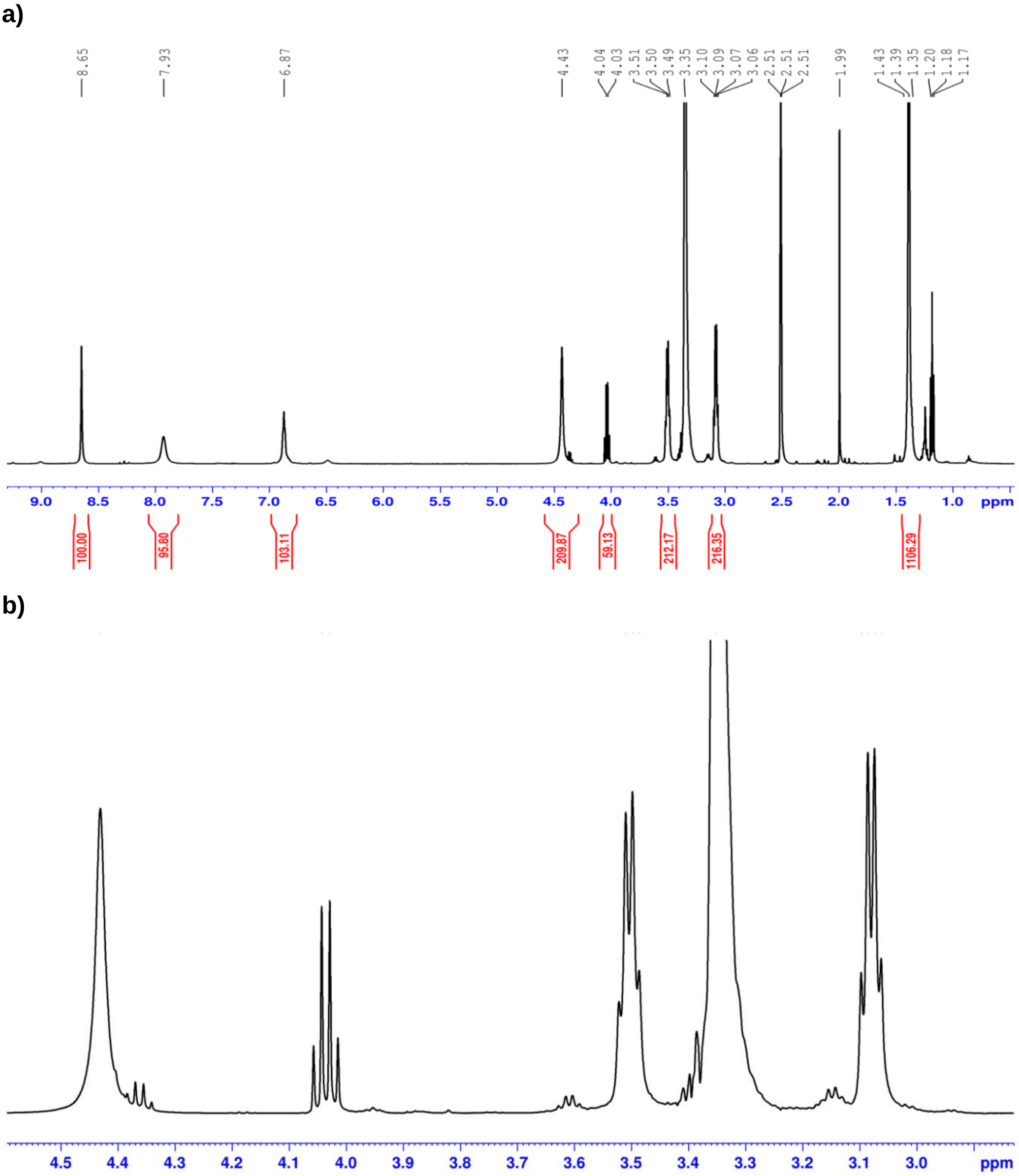

Fig. S3. ^{1}H NMR spectrum of t-butyl(2-hydrazinecarbothioamide ethyl)carbamate **(3)**. [Conditions: 500 MHz, DMSO-d_6]. **(A)** is the entire spectrum, **(B)** is the zoom-in of the 1.0-4.5 ppm region.

a)

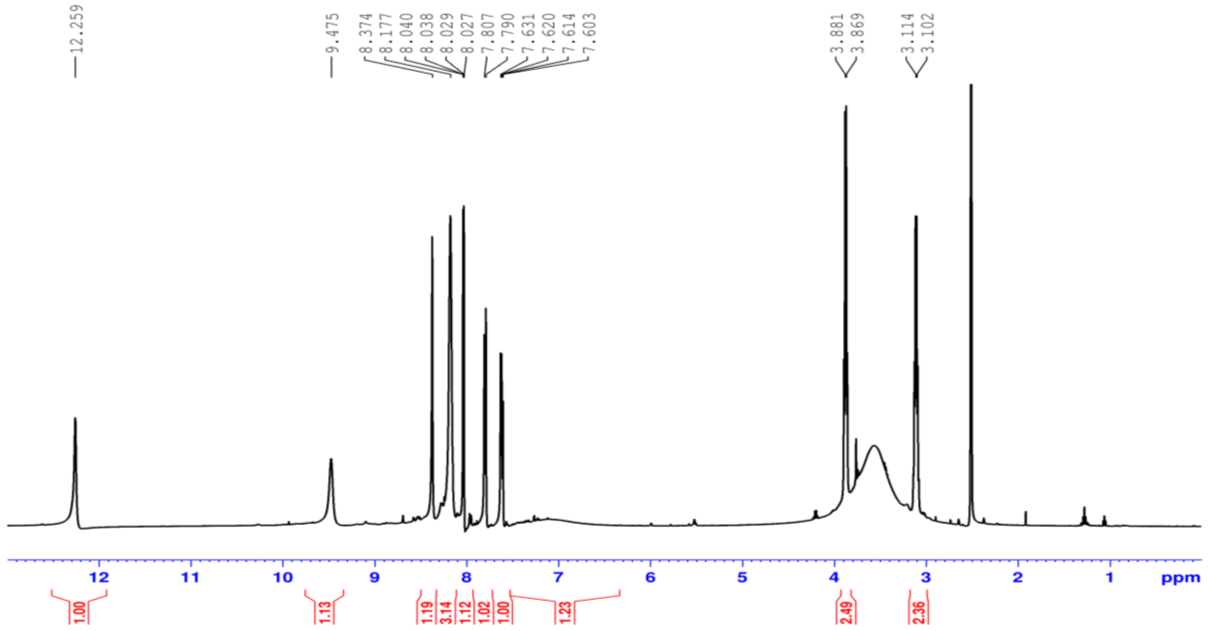

b)

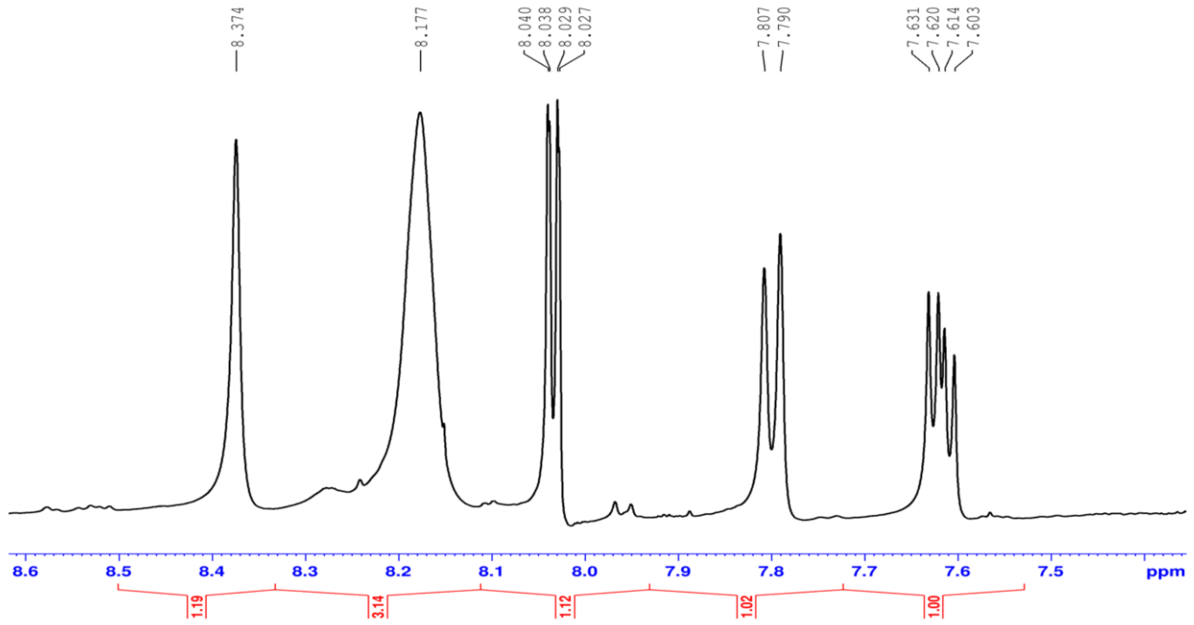

Fig. S4. ^{1}H NMR spectrum of NEtTrp·2HCl **(4)**. [Conditions: 500 MHz, DMSO-d_6]. **(A)** is the entire spectrum, **(B)** is the zoom-in of the 7.0-8.5 ppm region.

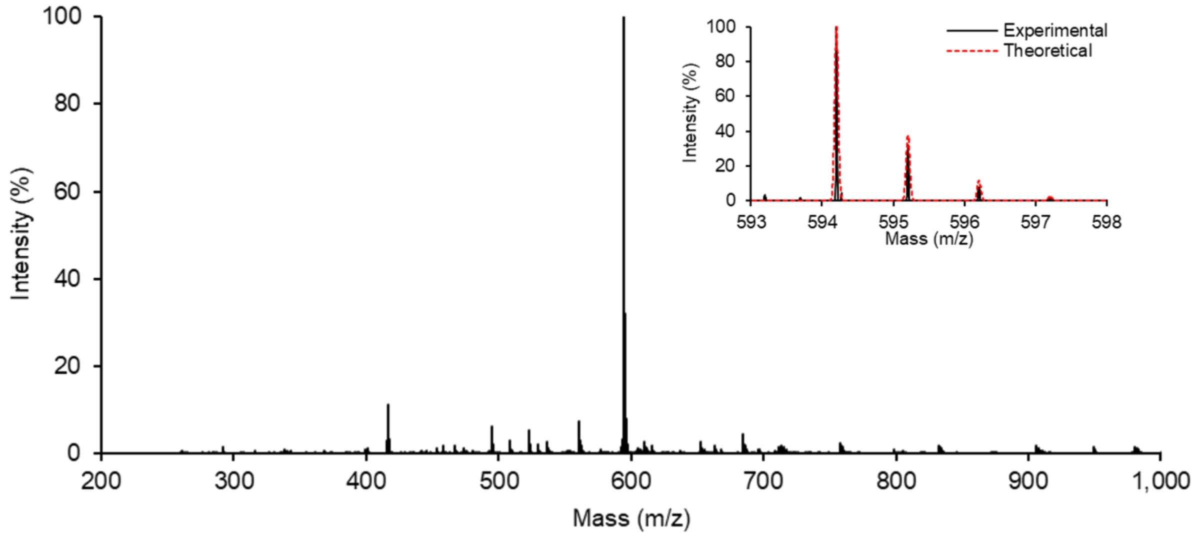

Fig. S5. ESI-MS (positive ion mode) of DefNEtTrp **(6)**: *m/z* 594.23, $\{(H^+)[C_{30}H_{27}N_9O_3S]\}^+$ (H^+ adduct of DefNEtTrp). The measured data is in black and the theoretical overlay is in red.

a)

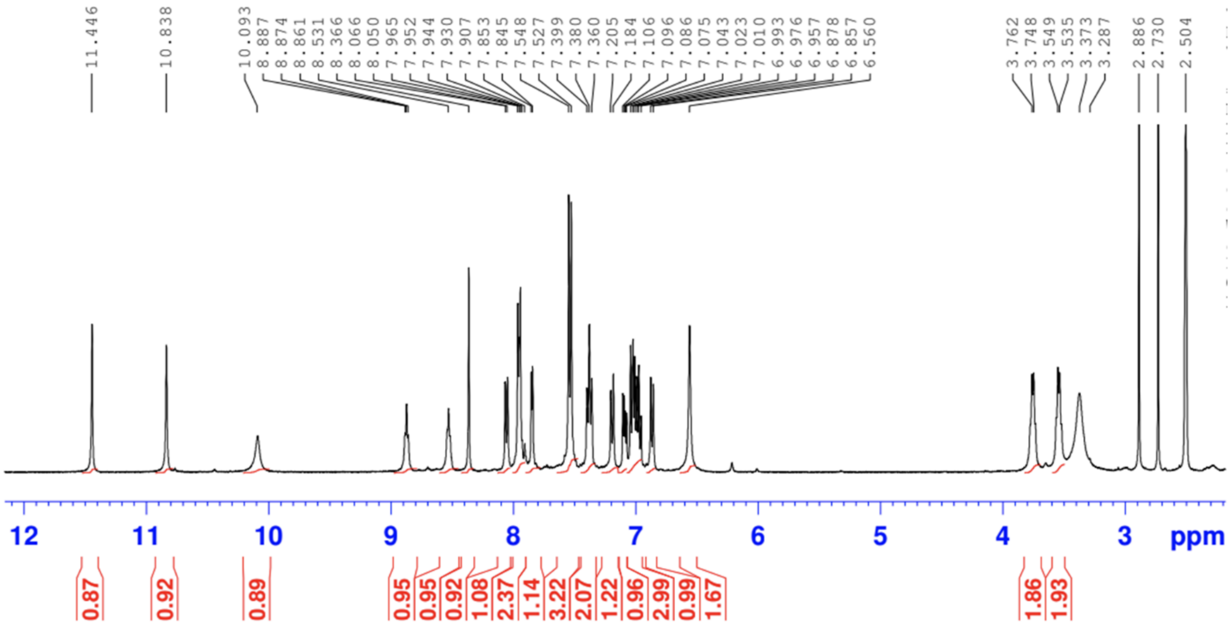

b)

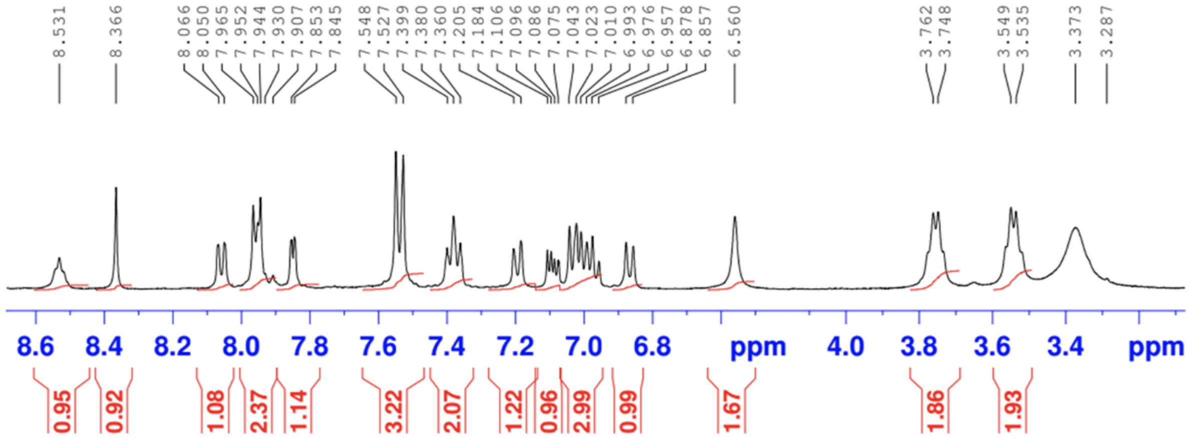

Fig. S6. ^{1}H NMR spectrum of DefNEtTrp **(6)**. [Conditions: 500 MHz, DMSO-d_6]. **(A)** is the entire spectrum, **(B)** is the zoom-in of the 3.2-8.6 ppm region.

a)

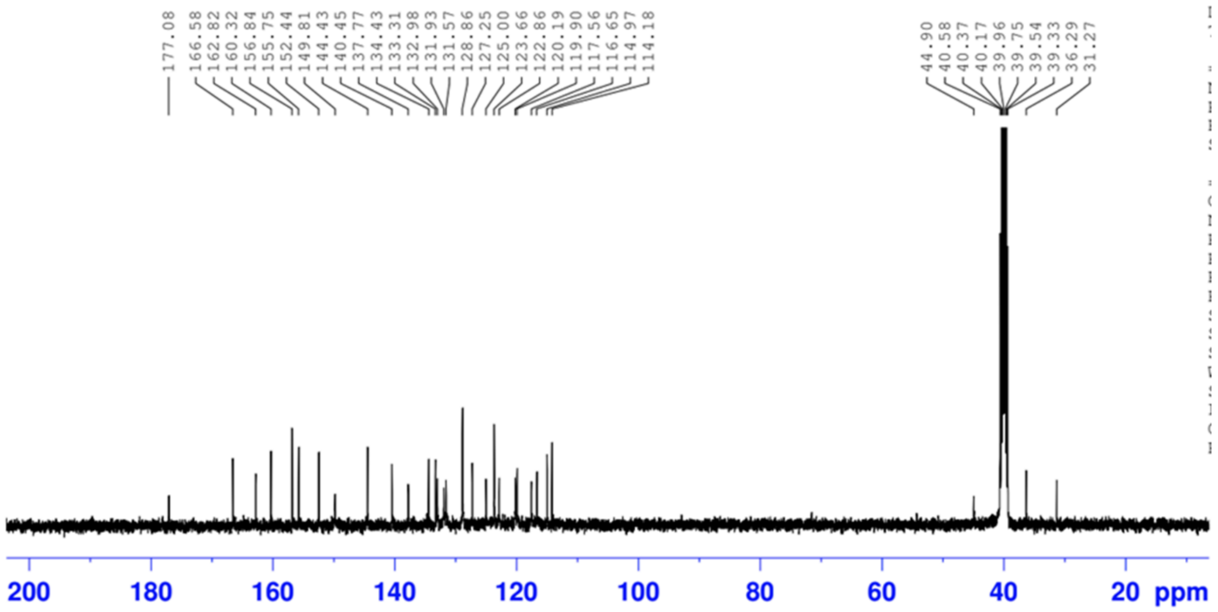

b)

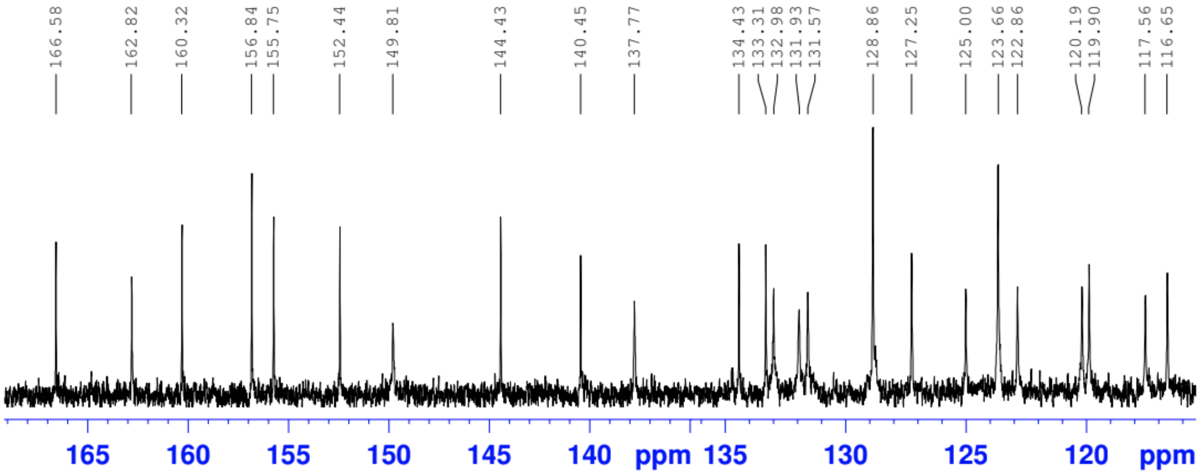

Fig. S7. ^{13}C NMR spectrum of DefNEtTrp **(6)**. [Conditions: 500 MHz, DMSO-d_6]. **(A)** is the entire spectrum, **(B)** is the zoom-in of the 115-170 ppm region.

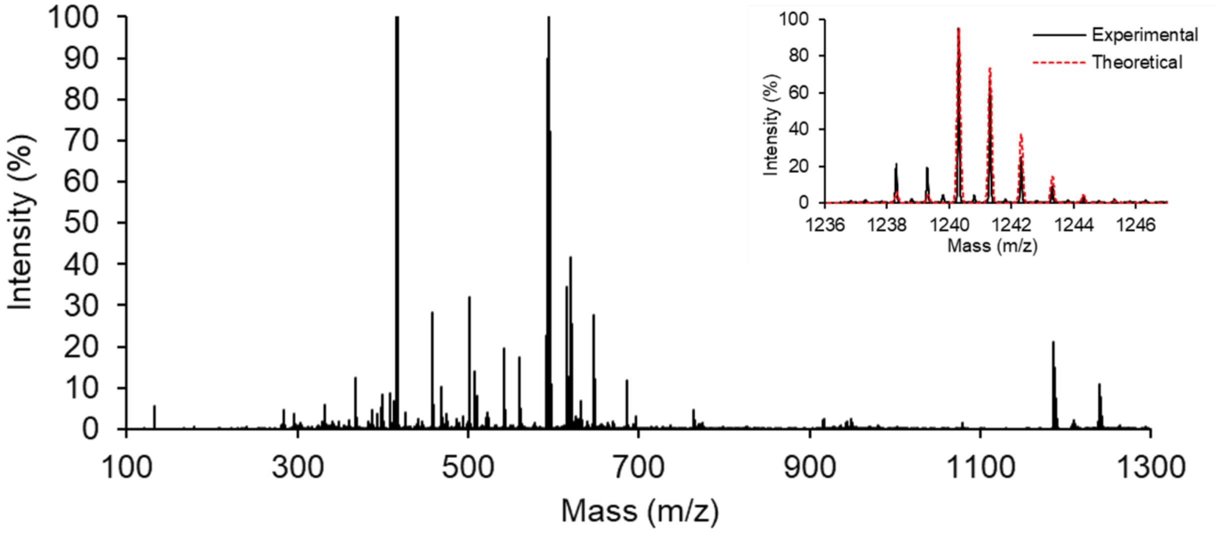

Fig. S8. ESI-MS (positive ion mode) of Fe(DefNetTrp)$_2$ **(7)**: *m/z* 1240.41, $\{2H^+ + [C_{60}H_{50}N_{18}O_6S_2Fe(III)]\}^+$. The measured data is in black and the theoretical overlay is in red.

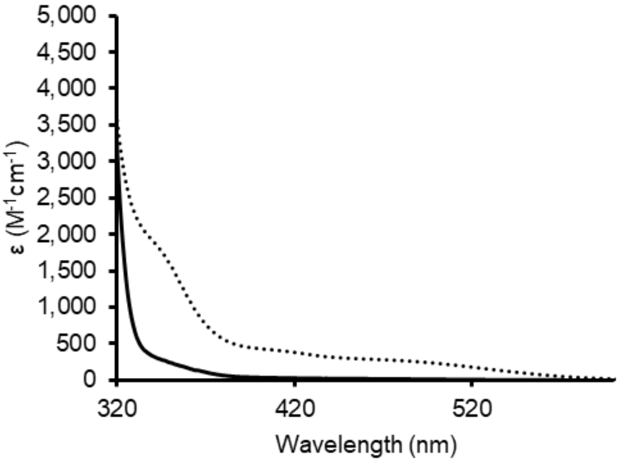

Fig. S9. UV-vis spectra for Deferasirox (black line) and Fe(Deferasirox)$_2$ **(9)** (dotted line) collected at pH 7.4 (50:50 (v/v) DMSO:H_2O solution (0.1 M Tris, 0.1 M NaCl). The extinction coefficient (ε) values are reported based on Deferasirox concentration.

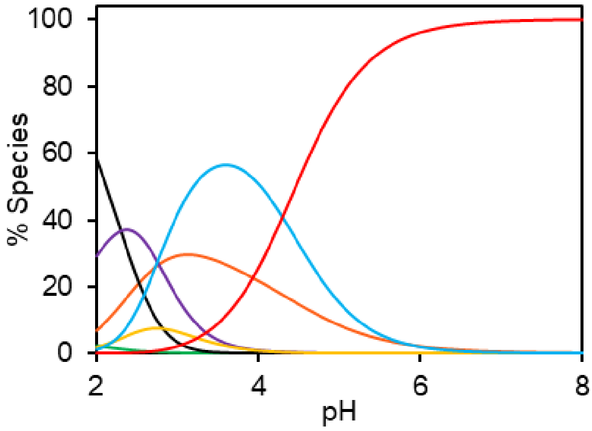

Fig. S10 The pH dependent aqueous speciation model for the interaction of 50 μM Fe(III) and fully deprotonated Def^{3-} and Trp^{-} at 100 μM each. This model only takes homoleptic compounds into consideration because that is the only speciation data available: (black) $[Fe(HDef)]^{+}$; (purple) [Fe(Def)] ; (yellow) $[Fe(HDef)_2]^{-}$; (blue) $[Fe(HDef)(Def)]^{2-}$; (red) $[Fe(Def)_2]^{3-}$; (orange) $[Fe(HTrp)_2]^{+}$; and (green) $[Fe(Trp)]^{2+}$.

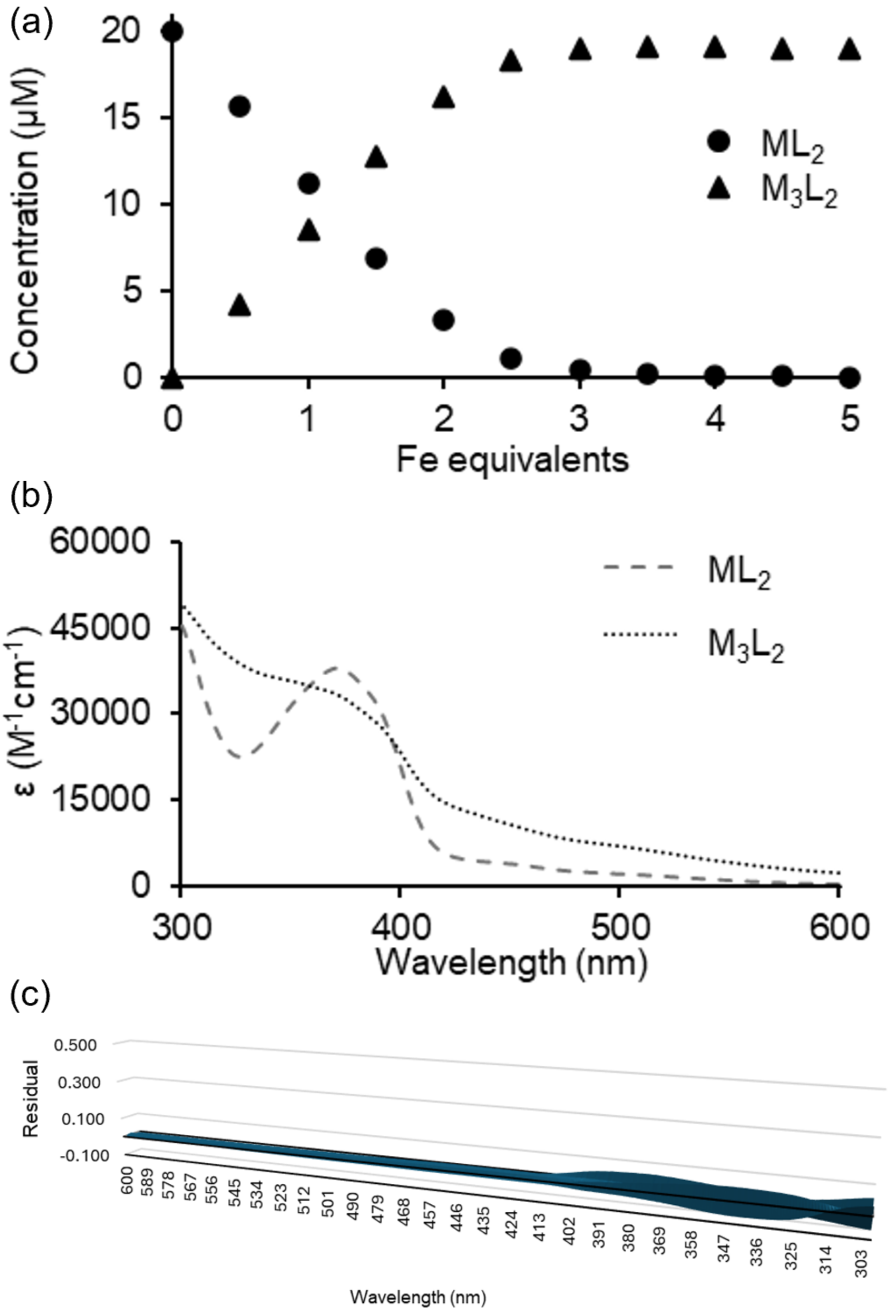

Fig. S11. Formation studies of $Fe_3(DefNEtTrp)_2$ **(8)** from the reaction of $Fe(DefNEtTrp)_2$ **(7)** and varying concentrations of Fe(III) up to five mole equivalents. The reactions were monitored by UV-vis spectroscopy over the 300 to 600 nm wavelength range and the data were deconvoluted with the ReactLab Equilibria software. **(A)** Species concentration profile throughout the Fe(III) titration. **(B)** Final species spectra for $Fe(DefNEtTrp)_2$ **(7)** and $Fe_3(DefNEtTrp)_2$ **(8)** determined by ReactLab Equilibria. **(C)** The residuals plot from fitting the reaction data.

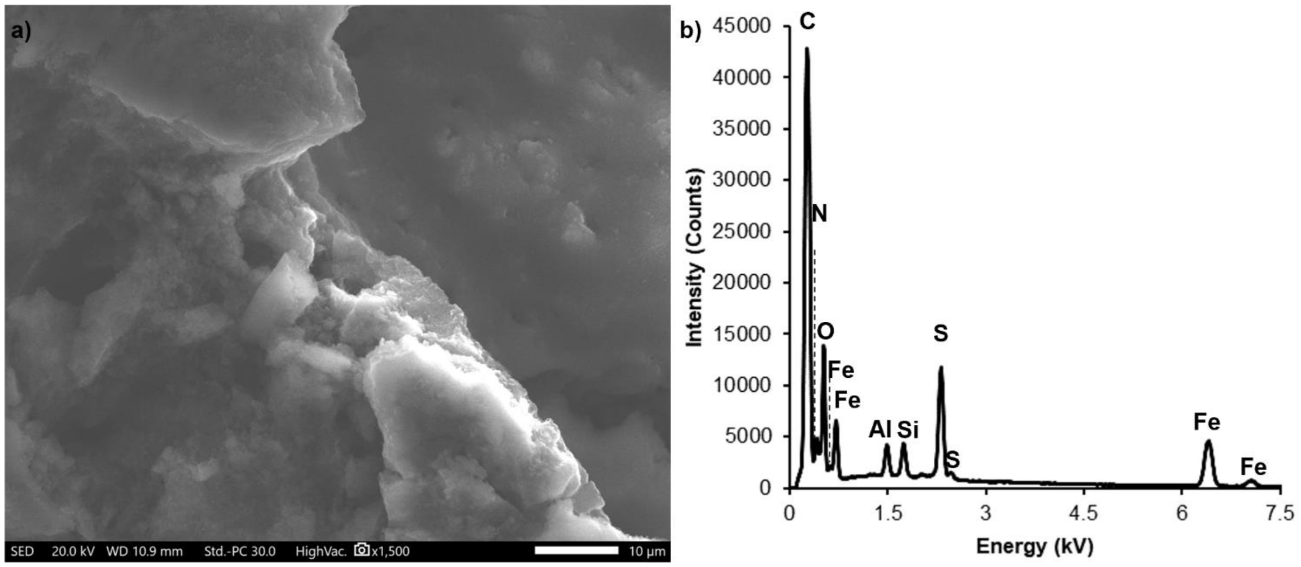

Fig. S12. Characterization of the $Fe_3(DefNEtTrp)_2$ **(8)** compound. Scanning electron microscopy image of the compound (a). Energy-dispersive X-ray spectrum displaying the elements present in the compound. Trace amounts of Al and Si are observed that are background contaminants and are not part of the compound composition.

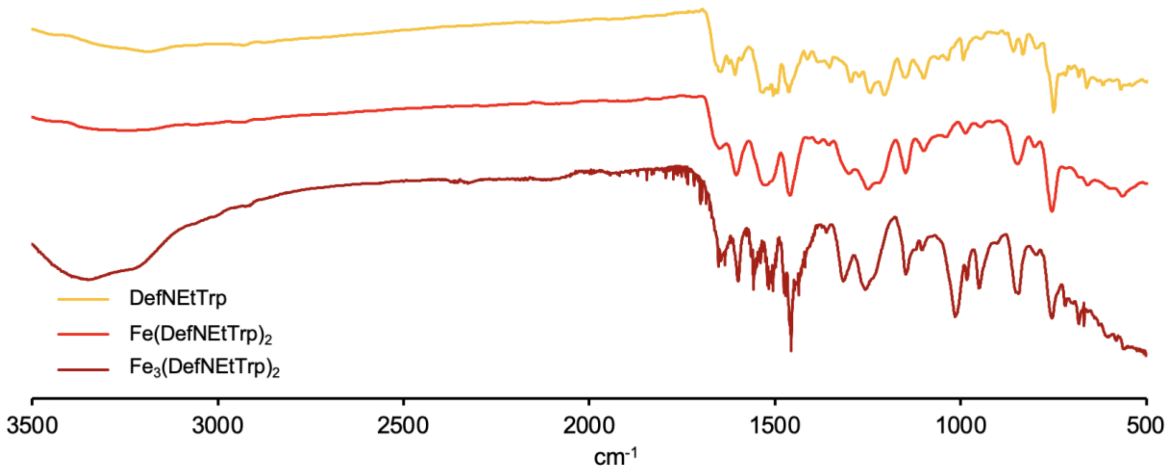

Fig. S13. FT-IR spectra of DefNEtTrp **(6)**, $Fe(DefNEtTrp)_2$ **(7)**, and $Fe_3(DefNEtTrp)_2$**(8)**.

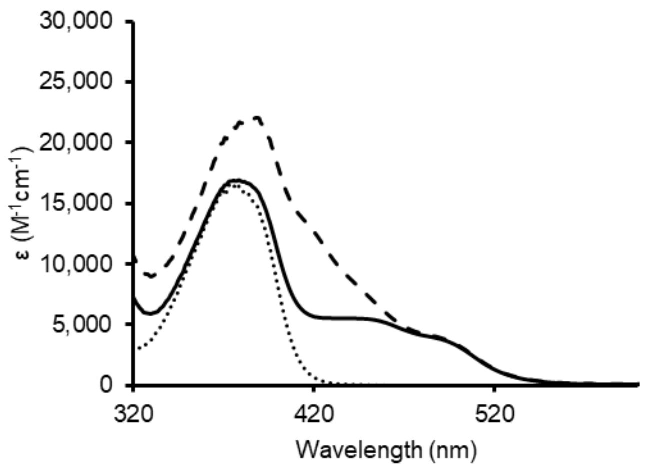

Fig. S14. UV-vis spectra for Triapine (dotted line), Fe(Trp) **(10)** (pH 2; dashed line) and Fe(Triapine)$_2$ **(11)** (pH 8, line). The extinction coefficient (ε) values are reported based on Triapine concentration.

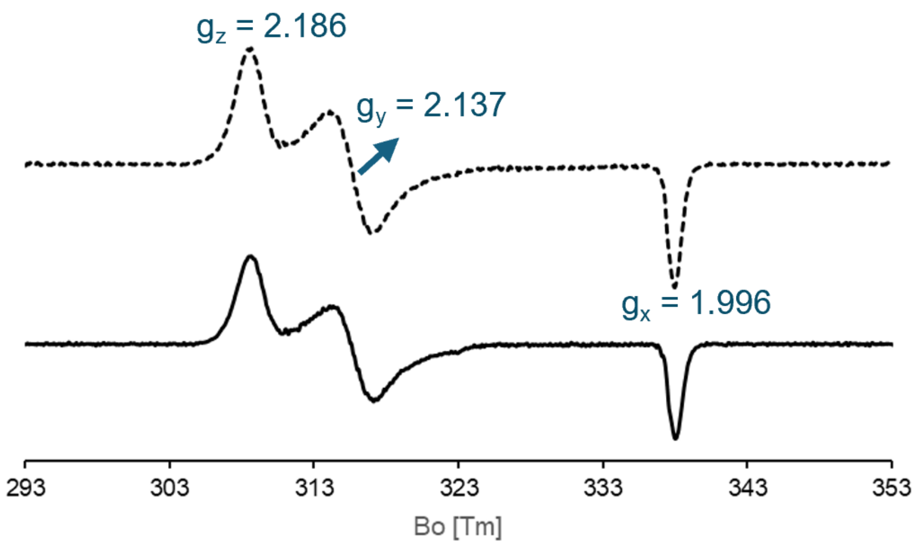

Fig. S15. The EPR spectra for Fe(Trp) **(10)** (pH 2, line) and Fe(Triapine)$_2$ **(11)** (pH 8, dotted line). Experimental conditions: microwave frequency, 9.441 GHz; microwave power, 200 μW; magnetic field modulation amplitude, 0.5 mT for low-spin Fe(III) (S = 1/2) detection, temperature: 77 K.

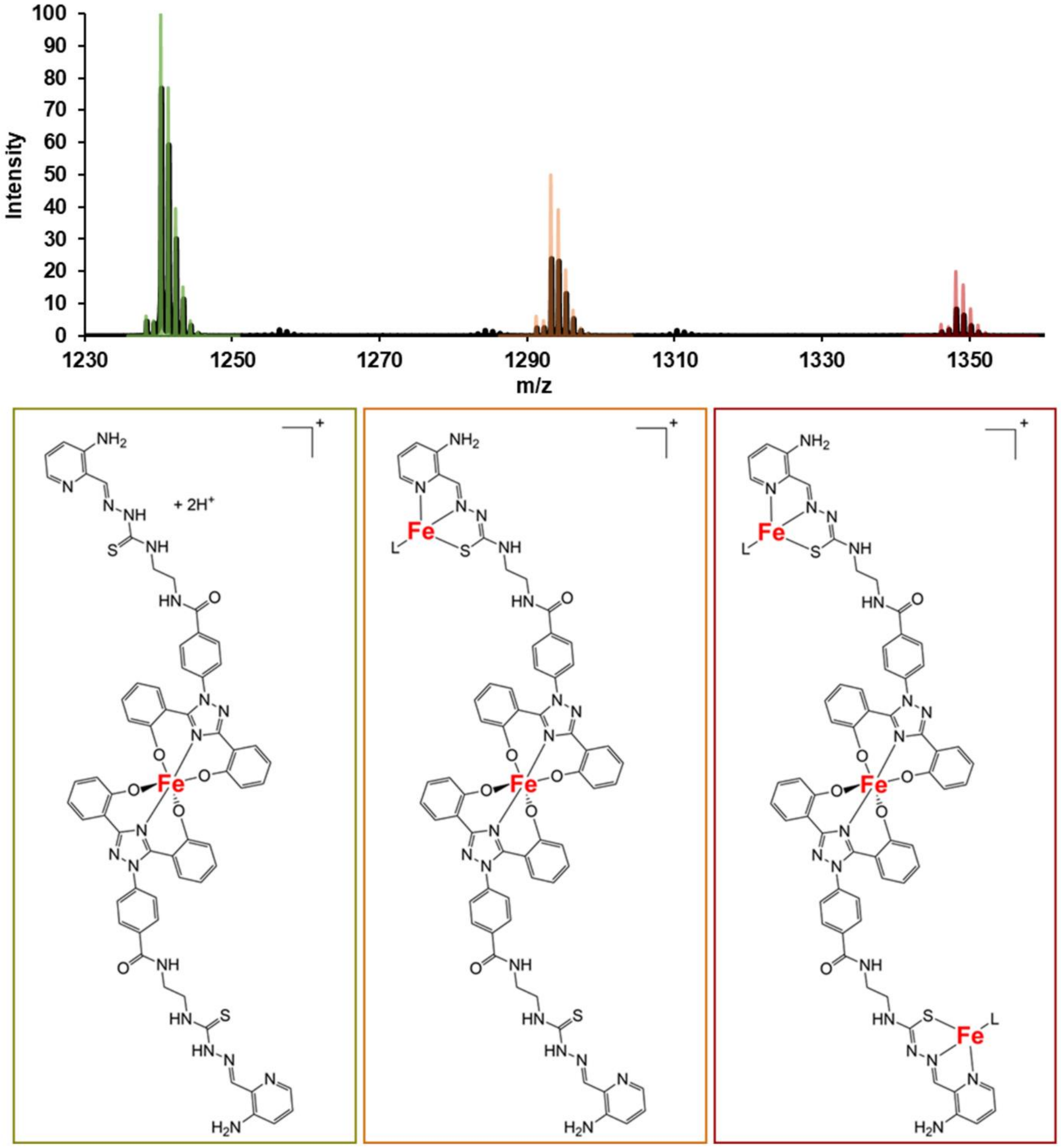

Fig. S16. MALDI-TOF MS (positive ion mode): *m/z* 1240.41, $\{(H^+)_2[C_{60}H_{50}N_{18}O_6S_2Fe]\}^+$ ($Fe(DefNetTrp)_2$) **(7)**; *m/z* 1293.32, $\{C_{60}H_{49}N_{18}O_6S_2Fe_2\}^+$ (($Fe_2(DefNetTrp)_2$); *m/z* 1348.10, $\{C_{60}H_{48}N_{18}O_6S_2Fe_3\}^+$ ($Fe_3(DefNetTrp)_2$) **(8)** (in the MALDI TOF source, two of the Fe ions in this species are reduced to Fe(II)). The measured data is in black, and the theoretical overlays for $Fe(DefNEtTrp)_2$ **(7)**, $Fe_2(DefNEtTrp)_2$, and $Fe_3DefNEtTrp)_2$ **(8)** are in green, orange, and red, respectively. The proposed structures for $Fe(DefNEtTrp)_2$ **(7)**, $Fe_2(DefNEtTrp)_2$, and $Fe_3(DefNEtTrp)_2$ **(8)** are boxed in green, orange, and red, respectively. Note that ligands L is not detected by the MALDI-TOF instrument but as discussed in the manuscript it is expected that the coordination number would be 6 and that aqua or hydroxo ligands would satisfy the remaining coordination sites.

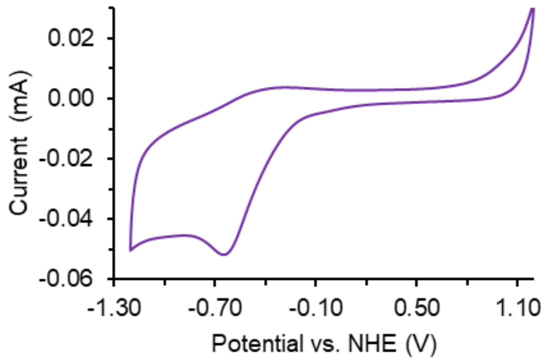

Fig. S17. Cyclic voltammogram of 6 mM Fe(Deferasirox)$_2$ **(9).**

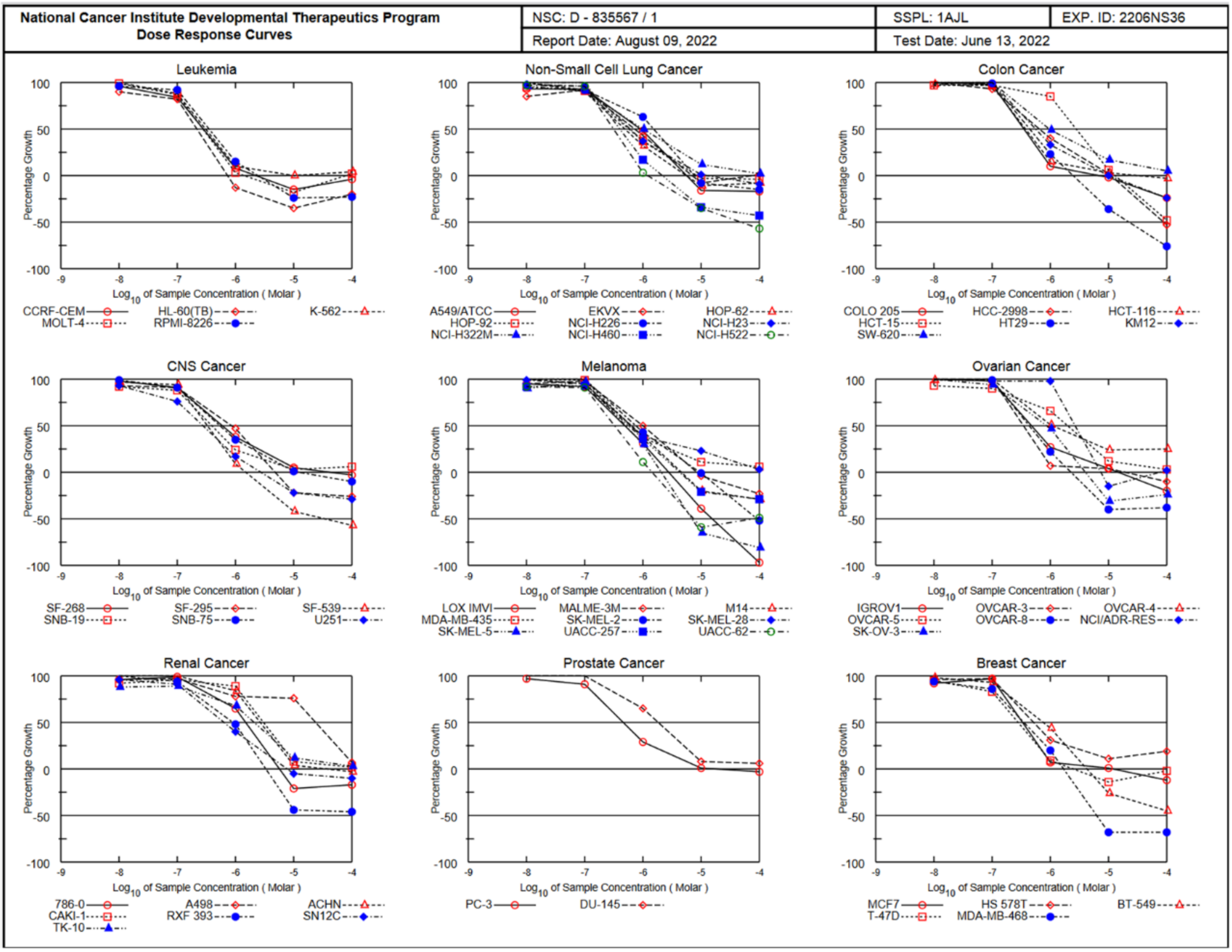

Fig. S18. Dose response curves of DefNEtTrp **(6)** against the nine different panels of cancer cell lines in the NCI-60 five dose screen.

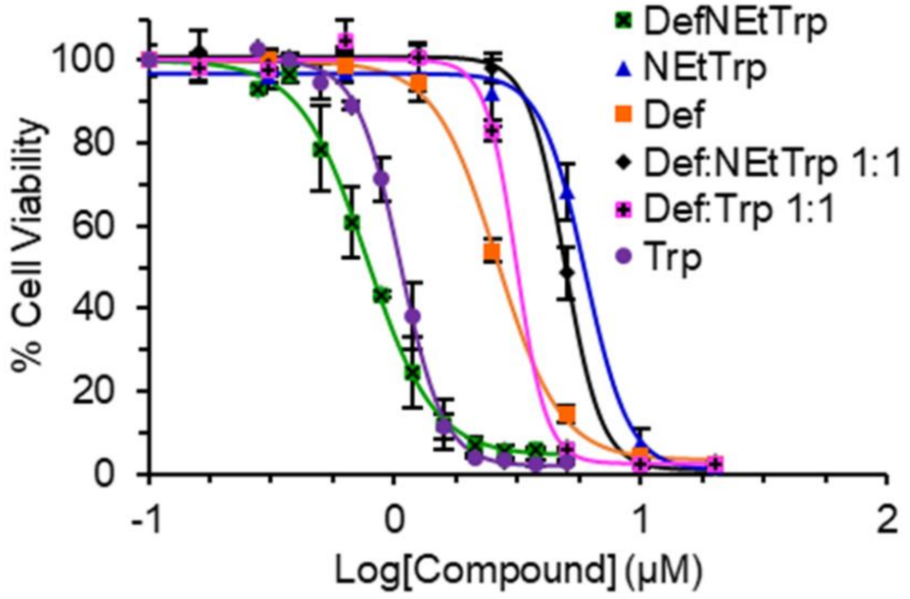

Fig. S19. Dose response curves for DefNEtTrp **(6)** (green), NEtTrp **(4)** (blue), Def **(5)** (orange), Trp (purple), 1:1 combination of Def and NEtTrp (black), 1:1 combination Def and Trp,(pink) against the Jurkat Leukemia cell line for 72 h.

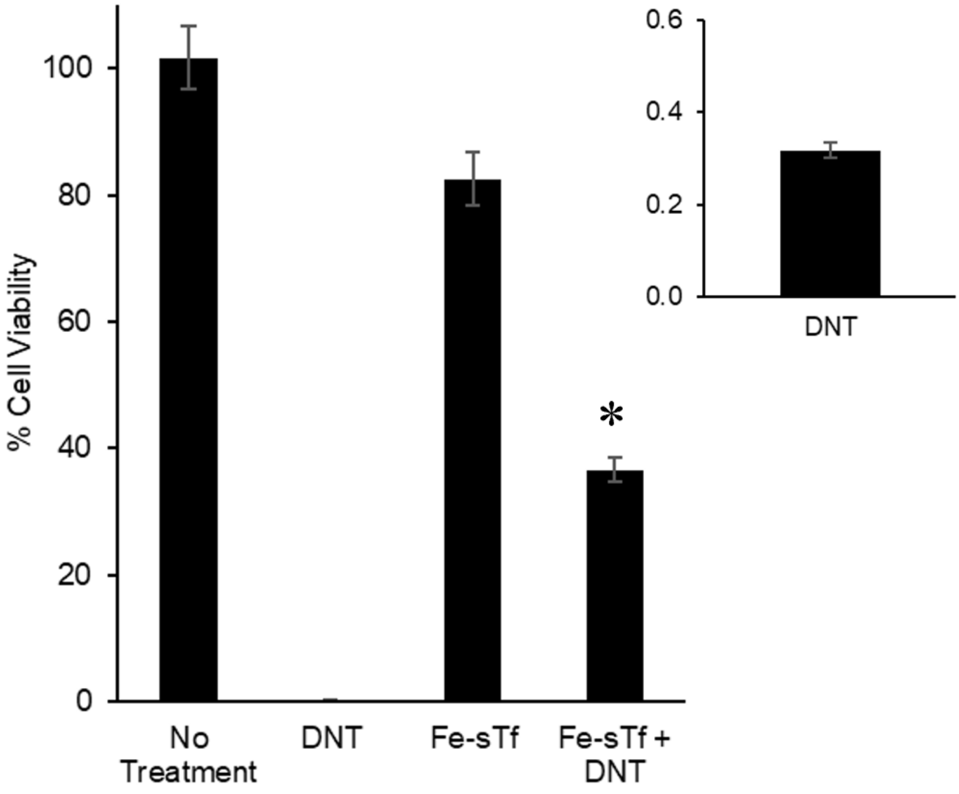

Fig. S20. Observed changes in the % cell viability of Jurkat cells presupplemented with 30 μM Fe(III)-saturated serum transferrin (Fe_2-STf-$(CO_3)_2$) (pH 7.4) for two hours and then treated for 72 h with 2 μM DefNEtTrp (DNT) **(6)** or media alone. * p-value < 0.01 vs the corresponding non-supplemented Fe group.

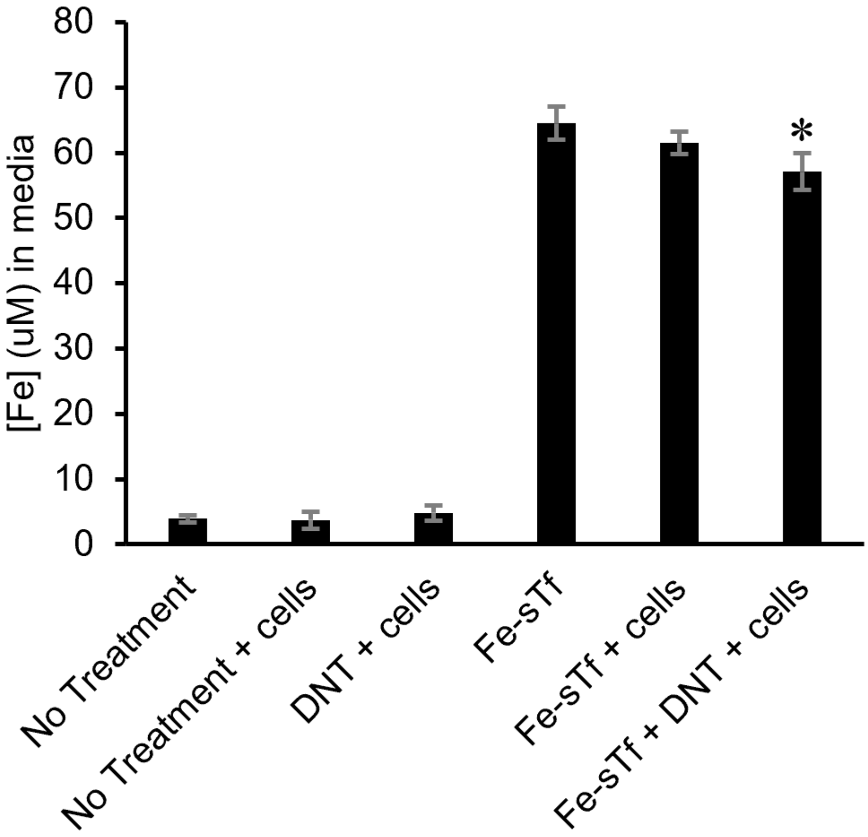

Fig. S21. Observed changes in the % cell viability of Jurkat cells presupplemented with 30 μM Fe(III)-saturated serum transferrin (Fe_2-STf-$(CO_3)_2$) (pH 7.4) for two hours and then treated for 72 h with 2 μM DefNEtTrp (DNT) **(6)** or media alone. * p-value < 0.01 vs the corresponding non-supplemented Fe group.

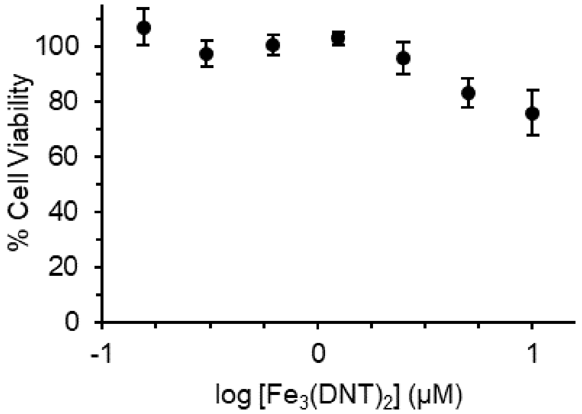

Fig. S22. Dose response curve for Fe_3(DefNEtTrp)$_2$ **(8)** against Jurkat cells for 72 h.

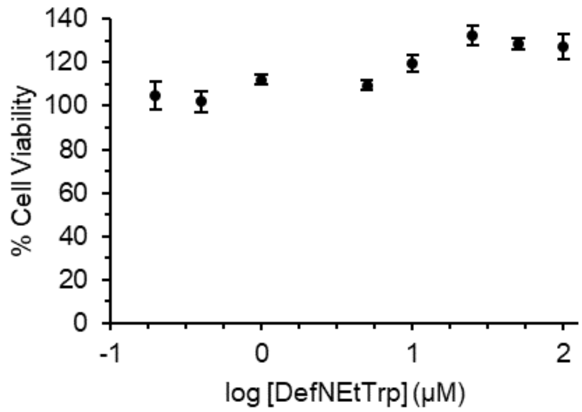

Fig. S23. Dose response curve for DefNEtTrp **(6)** against the MRC-5 noncancer lung cell line for 72 h.

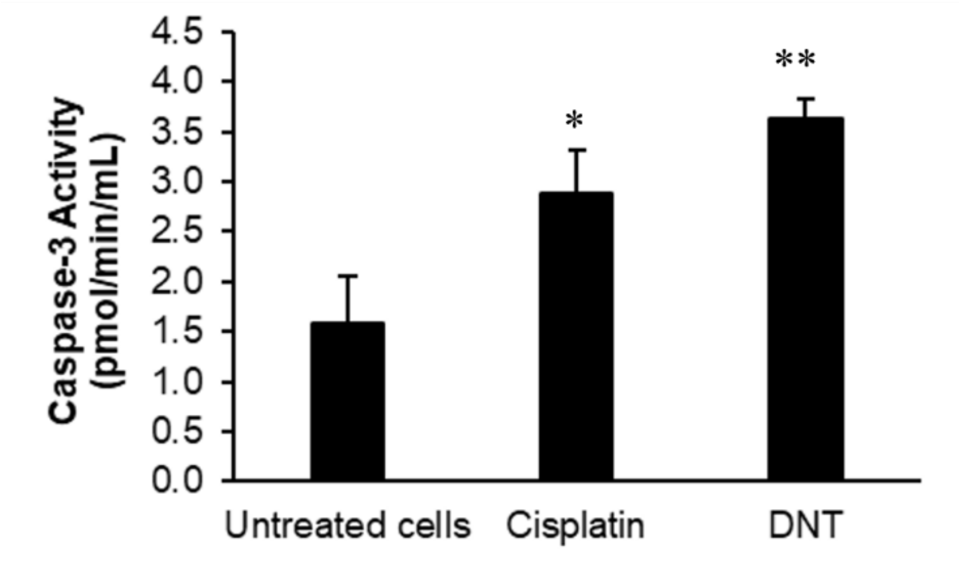

Fig. S24. Caspase-3 apoptosis activity assay in Jurkat cells with media alone (untreated), 20 μM cisplatin, or 2 μM DefNEtTrp **(6)** (DNT). * p-value < 0.05; ** p-value < 0.01 vs the untreated group.

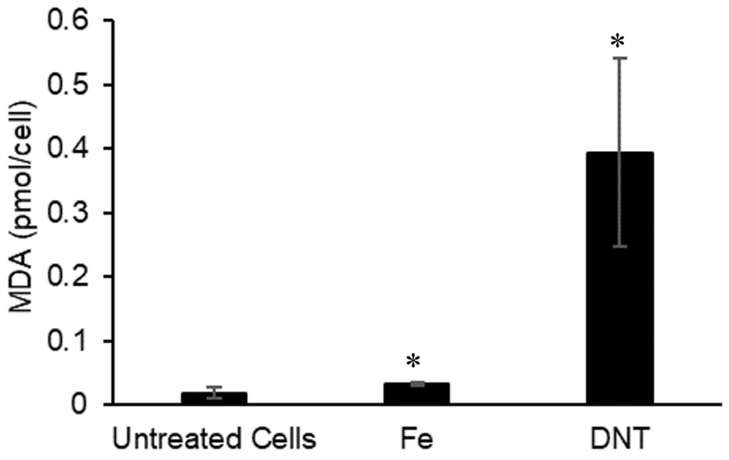

Fig. S25. Ferroptosis activity assay following the formation of MDA after 72 h in Jurkat cells with media alone (untreated), 25 μM Fe(citrate)$_2$ (Fe), or 2 μM DefNEtTrp **(6)** (DNT). * p-value < 0.05 vs the corresponding untreated group.

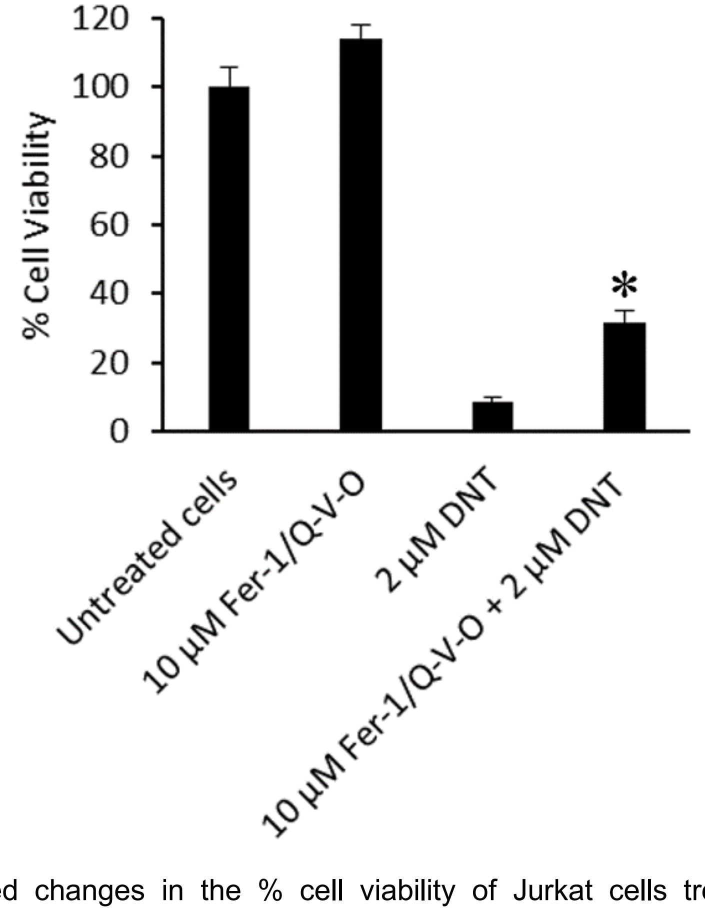

Fig. S26. Observed changes in the % cell viability of Jurkat cells treated with 10 μM Ferrastatin-1/Q-VD-OPh (Fer-1/Q-V-O) in combination and then treated for 72 h with 2 μM DefNEtTrp **(6)** (DNT) or media alone. * p-value < 0.01 vs the corresponding 2 μM DefNEtTrp without the Fer-1/Q-V-O combination.

3. Supporting Tables

Table S1. Crystal and structure refinement for NEtTrp·2HCl **(4)**.

Empirical formula	$C_9H_{16}N_6S$, Cl_2
Formula weight	311.24
Temperature	293 K
Wavelength	1.54184 Å
Crystal system, space group	Triclinic, P-1
Unit cell dimensions	a = 6.3575(10) Å, α = 83.360(10) º<br>b = 9.9212(10) Å, β = 80.133(10) º<br>c = 11.7282(10) Å, γ = 86.844(10) º
Volume	723.445(15) $Å^3$
Z, calculated density	2, 1.429 g/cm^3
Absorption coefficient	5.338 mm^{-1}
F(000)	324.0
Crystal size	0.232 × 0.157 × 0.041 mm
Theta range for data collection	7.696 to 137.7º
Limiting indices	$-7 \leq h \leq 7$, $-11 \leq k \leq 11$, $-14 \leq l \leq 14$
Reflections collected/unique	13912/2664 (R_{int} = 0.0564)
Completeness to theta = 68.850°	99.14%
Absortion	Multi-scan
Max. And min. transmission	0.097 and 1.000
Refinement method	Full-matrix least squares on F^2
Data/restraints/parameters	2264/0/170
Goodness-of-fit on F^2<br>Final R indices [I > 2sigma(I)]	1.138<br>R_1 = 0.0404, wR_2 = 0.1243
R indices (all data)	R_1 = 0.0417, wR_2 = 0.1257
Largest diff. peak and hole	0.38 and -0.27 e $Å^{-3}$

Table S2. A summary of the NCI-60 cancer cell line viability screen of DefNEtTrp **(6)** at the five-dose level for 48 h.

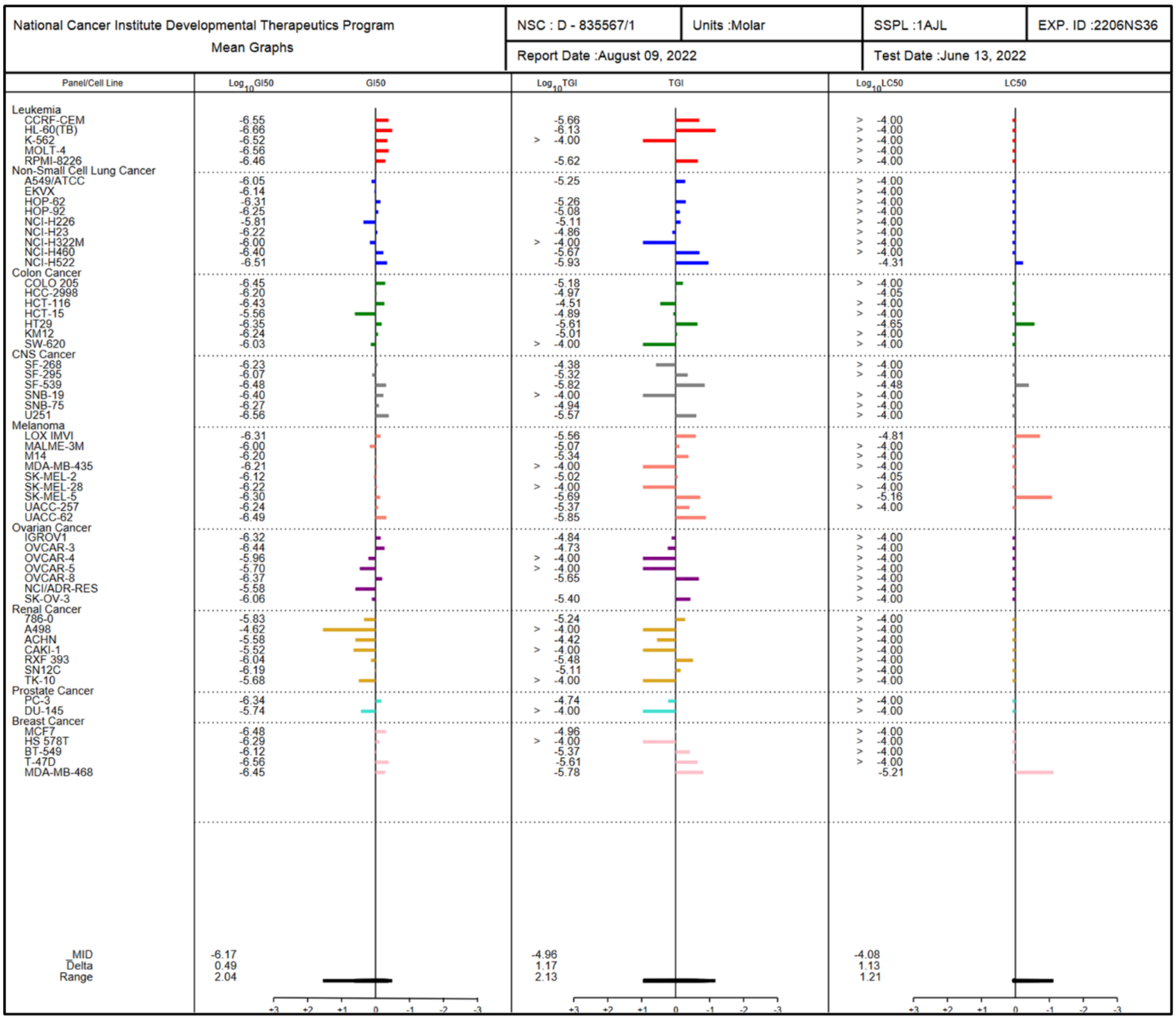

National Cancer Institute Developmental Therapeutics Program Mean Graphs	NSC : D - 835567/1	Units :Molar	SSPL :1AJL	EXP. ID :2206NS36
	Report Date :August 09, 2022		Test Date :June 13, 2022	

Panel/Cell Line	Log_{10}GI50	Log_{10}TGI	Log_{10}LC50
**Leukemia**			
CCRF-CEM	-6.55	-5.66	> -4.00
HL-60(TB)	-6.66	-6.13	> -4.00
K-562	-6.52	> -4.00	> -4.00
MOLT-4	-6.56		> -4.00
RPMI-8226	-6.46	-5.62	> -4.00
**Non-Small Cell Lung Cancer**			
A549/ATCC	-6.05	-5.25	> -4.00
EKVX	-6.14		> -4.00
HOP-62	-6.31	-5.26	> -4.00
HOP-92	-6.25	-5.08	> -4.00
NCI-H226	-5.81	-5.11	> -4.00
NCI-H23	-6.22	-4.86	> -4.00
NCI-H322M	-6.00	> -4.00	> -4.00
NCI-H460	-6.40	-5.67	> -4.00
NCI-H522	-6.51	-5.93	-4.31
**Colon Cancer**			
COLO 205	-6.45	-5.18	> -4.00
HCC-2998	-6.20	-4.97	-4.05
HCT-116	-6.43	-4.51	> -4.00
HCT-15	-5.56	-4.89	> -4.00
HT29	-6.35	-5.61	-4.65
KM12	-6.24	-5.01	> -4.00
SW-620	-6.03	> -4.00	> -4.00
**CNS Cancer**			
SF-268	-6.23	-4.38	> -4.00
SF-295	-6.07	-5.32	> -4.00
SF-539	-6.48	-5.82	-4.48
SNB-19	-6.40	> -4.00	> -4.00
SNB-75	-6.27	-4.94	> -4.00
U251	-6.56	-5.57	> -4.00
**Melanoma**			
LOX IMVI	-6.31	-5.56	-4.81
MALME-3M	-6.00	-5.07	> -4.00
M14	-6.20	-5.34	> -4.00
MDA-MB-435	-6.21	> -4.00	> -4.00
SK-MEL-2	-6.12	-5.02	-4.05
SK-MEL-28	-6.22	> -4.00	> -4.00
SK-MEL-5	-6.30	-5.69	-5.16
UACC-257	-6.24	-5.37	> -4.00
UACC-62	-6.49	-5.85	
**Ovarian Cancer**			
IGROV1	-6.32	-4.84	> -4.00
OVCAR-3	-6.44	-4.73	> -4.00
OVCAR-4	-5.96	> -4.00	> -4.00
OVCAR-5	-5.70	> -4.00	> -4.00
OVCAR-8	-6.37	-5.65	> -4.00
NCI/ADR-RES	-5.58		> -4.00
SK-OV-3	-6.06	-5.40	> -4.00
**Renal Cancer**			
786-0	-5.83	-5.24	> -4.00
A498	-4.62	> -4.00	> -4.00
ACHN	-5.58	-4.42	> -4.00
CAKI-1	-5.52	> -4.00	> -4.00
RXF 393	-6.04	-5.48	> -4.00
SN12C	-6.19	-5.11	> -4.00
TK-10	-5.68	> -4.00	> -4.00
**Prostate Cancer**			
PC-3	-6.34	-4.74	> -4.00
DU-145	-5.74	> -4.00	> -4.00
**Breast Cancer**			
MCF7	-6.48	-4.96	> -4.00
HS 578T	-6.29	> -4.00	> -4.00
BT-549	-6.12	-5.37	> -4.00
T-47D	-6.56	-5.61	> -4.00
MDA-MB-468	-6.45	-5.78	-5.21
MID	-6.17	-4.96	-4.08
Delta	0.49	1.17	1.13
Range	2.04	2.13	1.21

Table S3. The calculated average 50% growth inhibition (GI_{50}) of Triapine and DefNEtTrp in μM compound concentration.

Cancer cell line type	Trp GI_{50} (μM)	DNT GI_{50} (μM)
Leukemia	1.57	0.25
Non-Small Cell Lung	12.1	0.46
Colon	1.47	0.55
CNS	0.57	0.33
Melanoma	3.1	0.49
Ovarian	2.57	0.65
Renal	0.37	2.31
Prostate	5.99	0.78
Breast	4.45	0.26

Table S4. Evaluation of the hemolytic activity of the compounds Trp, NEtTrp (4), Def (5), and DefNEtTrp (6). Positive control: melittin. D_{max} is the maximal percentage of hemolysis measured.

Compound	Concentration in µg/mL; µM	D_{max} (%)
Trp	32.0; 164	9.45
NEtTrp	32.0; 103	6
Def	32.0; 85.7	5.3
DefNEtTrp	32.0; 50.3	5.3
Melittin	14.2; 5	>95

4. References

(1) Tinoco, A. D.; Peterson, C. W.; Lucchese, B.; Doyle, R. P.; Valentine, A. M. On the evolutionary significance and metal-binding characteristics of a monolobal transferrin from Ciona intestinalis. *Proc. Natl. Acad. Sci. U. S. A.* **2008**, *105* (9), 3268-3273. DOI: 10.1073/pnas.0705037105

(2) DesMarteau, D.; Changqing, l. Syntheses and lipophilicity measurement of N α/ N-terminus-1,1-dihydroperfluoroalkylated α-amino acids and small peptides. *J. Fluor. Chem.* **2007**, *128*, 1326-1334. DOI: 10.1016/j.jfluchem.2007.07.003

(3) Steinhauser, S.; Heinz, U.; Bartholomä, M.; Weyhermüller, T.; Nick, H.; Hegetschweiler, K. Complex formation of ICL670 and related ligands with Fe(III) and Fe(II). *Eur. J. Inorg. Chem.* **2004**, *2004* (21), 4177-4192. DOI: 10.1002/ejic.200400363

(4) Enyedy, E. A.; Primik, M. F.; Kowol, C. R.; Arion, V. B.; Kiss, T.; Keppler, B. K. Interaction of triapine and related thiosemicarbazones with iron(III)/(II) and gallium(III): A comparative solution equilibrium study. *Dalton Trans.* **2011**, *40* (22), 5895-5905. DOI: 10.1039/c0dt01835j

(5) Stefánsson, A. Iron(III) hydrolysis and solubility at 25 °C. *Environ. Sci. Technol.* **2007**, *41* (17), 6117-6123. DOI: 10.1021/es070174h

(6) Viollier, E.; Inglett, P. W.; Hunter, K.; Roychoudhury, A. N.; Van Cappellen, P. The ferrozine method revisited: Fe(II)/Fe(III) determination in natural waters. *Appl. Geochem.* **2000**, *15* (6), 785-790. DOI: 10.1016/S0883-2927(99)00097-9

(7) Schlabach, M. R.; Bates, G. W. The synergistic binding of anions and Fe3+ by transferrin. Implications for the interlocking sites hypothesis. *J. Biol. Chem.* **1975**, *250* (6), 2182-2188. DOI: 10.1016/S0021-9258(19)41699-2
